# Macrophage‐derived HMGB1 is dispensable for tissue fibrogenesis

**DOI:** 10.1096/fba.2018-00035

**Published:** 2019-02-12

**Authors:** Jean Personnaz, Enzo Piccolo, Maxime Branchereau, Aveline Filliol, Romain Paccoud, Elsa Moreau, Denis Calise, Elodie Riant, Pierre Gourdy, Christophe Heymes, Robert F. Schwabe, Cédric Dray, Philippe Valet, Jean‐Philippe Pradère

**Affiliations:** ^1^ Institut des Maladies Métaboliques et Cardiovasculaires, UMR 1048/I2MC, Institut National de la Santé et de la Recherche Médicale (INSERM), Université de Toulouse Toulouse France; ^2^ Department of Medicine Columbia University New York New York; ^3^ UMS006, Université de Toulouse, Institut National de la Santé et de la Recherche Médicale (INSERM) U1048, Institute of Cardiovascular and Metabolic Disease Toulouse France; ^4^ Service de Diabétologie, Maladies Métaboliques et Nutrition, CHU de Toulouse Toulouse France

**Keywords:** alarmin, DAMP, fibrosis, innate immunity

## Abstract

Alarmins and damage‐associated molecular patterns (DAMPs) are powerful inflammatory mediators, capable of initiating and maintaining sterile inflammation during acute or chronic tissue injury. Recent evidence suggests that alarmins/DAMPs may also trigger tissue regeneration and repair, suggesting a potential contribution to tissue fibrogenesis. High mobility group B1 (HMGB1), a bona fide alarmin/DAMP, may be released passively by necrotic cells or actively secreted by innate immune cells. Macrophages can release large amounts of HMGB1 and play a key role in wound healing and regeneration processes. Here, we hypothesized that macrophages may be a key source of HMGB1 and thereby contribute to wound healing and fibrogenesis. Surprisingly, cell‐specific deletion approaches, demonstrated that macrophage‐derived HMGB1 is not involved in tissue fibrogenesis in multiple organs with different underlying pathologies. Compared to control HMGB1^Flox^ mice, mice with macrophage‐specific HMGB1 deletion (HMGB1^ΔMac^) do not display any modification of fibrogenesis in the liver after CCL_4_ or thioacetamide treatment and bile duct ligation; in the kidney following unilateral ureter obstruction; and in the heart after transverse aortic constriction. Of note, even under thermoneutral housing, known to exacerbate inflammation and fibrosis features, HMGB1^ΔMac^ mice do not show impairment of fibrogenesis. In conclusion, our study clearly establishes that macrophage‐derived HMGB1 does not contribute to tissue repair and fibrogenesis.

**Abbreviations**
Acta2: alpha actin2
ALT: alanine transaminase
AST: aspartate transaminase
BDL: bile duct ligation
BW: body weight
CCL_4_: carbon tetrachloride
Col1a1: type1 alpha1 collagen
Col1a2: type1 alpha2 collagen
Col3a1: type3 alpha1 collagen
DAMPS: damage‐associated molecular patterns
DNA: deoxyribonucleic acid
Emr1: EGF‐like module containing mucin‐like hormone receptor‐like 1
FS: fractional shortening
GAPDH: glyceraldehyde‐3‐phosphate dehydrogenase
HMGB1: high mobility group box 1
HW/TL: heart weight to tíbia length ratio
IL‐18: interleukin 18
IL‐1β: interleukin 1β
LPS: lipopolysaccharide
LVEDD: left ventricular end‐diastolic dimension
LVESD: left ventricular end‐systolic dimension
PCR: polymerase chain reaction
PRR: pattern recognition receptor
RAGE: receptor for advanced glycation end products
TAA: thioacetamide
TAC: transverse aortic constriction
TLR: toll‐like receptor
TNFα: tumor necrosis factor α
UUO: unilateral ureteral obstruction
α‐SMA: α‐smooth muscle actin

## 1 INTRODUCTION

Sterile inflammation and regeneration are two biological processes that are tightly related to wound healing, but if uncontrolled could lead to scarring and fibrotic responses, contributing to the long term to organ loss of function.^1^, ^2^ Among cells involved in the modulation of these crucial functions, several types of macrophages play a central role in the clearance of dying/dead cells, and promote tissue repair by secreting a broad variety of molecules, such as cytokines, chemokines, or alarmins,^1^, ^2^, ^3^, ^4^ that either maintain the inflammation process and/or activate tissue myofibroblasts in charge of synthesizing extracellular matrix components to promote tissue architecture restoration.^2^, ^5^ Globally in the context of wound healing, immunostimulatory molecules originating either from macrophages (alarmins) or dying cells (such as damage‐associated molecular patterns [DAMPs]) have been characterized as both pro‐inflammatory but also as pro‐regenerating agents involved in progenitor cell proliferation and differentiation,^1^, ^3^, ^4^ thus potentially involve during chronic tissue injury, in inflammation and fibrosis processes which are now supported by several reports in the heart and lungs.^6^, ^7^ Among pro‐inflammatory and pro‐repair agents secreted by macrophages, high mobility group box 1 (HMGB1), also known for its alarmin and DAMP properties, has drawn a lot of attention as it is considered as an attractive target to treat acute and chronic inflammatory diseases^8^, ^9^, ^10^ and is also envisioned as a relevant biomarker of tissue injury^11^, ^12^ in humans. HMGB1 can reach the extracellular space, once released by necrotic epithelial cells and exerts its DAMPs function or when secreted by activated innate immune cells, where it mediates potent inflammatory effects.^10^, ^13^ Macrophages and other innate immune cells actively secrete HMGB1 upon an inflammatory challenge (interleukin 1β [IL1β], lipopolysaccharide [LPS], or tumor necrosis factor α [TNFα]) in vitro and in vivo^14^, ^15^, ^16^, ^17^ supporting a possible role of macrophage‐derived HMGB1 in tissue scarring. Recent publications documented a critical role of extracellular HMGB1 in regeneration and tissue repair processes in bone, muscle, or liver^18^, ^19^, ^20^, ^21^ suggesting a potential role in tissue fibrogenesis. Moreover, HMGB1 has been proposed to play a direct role in promoting fibrosis in the liver, lungs, kidney, and heart through different pathways such as inflammation or myofibroblast activation.^22^, ^23^, ^24^ However, in liver fibrosis, hepatocyte‐derived HMGB1 is likely not involved in fibrogenesis as shown in recent studies,^19^, ^20^ excluding any potential role of HMGB1 as a DAMP in liver scarring progression implying that non‐parenchymal cells, such as macrophages, could be the cellular source of HMGB1 during tissue fibrosis development.

To determine whether macrophage‐derived HMGB1 has a determinant role in fibrosis progression, we evaluated the impact of a macrophage‐specific HMGB1 genetic deletion on the liver, kidney, and cardiac fibrosis. Using very well‐established fibrosis models in three different organs, our anatomo‐pathological explorations revealed that unexpectedly, macrophage‐derived HMGB1 has no major role in fibrogenesis in mice.

## 2 MATERIALS AND METHODS

### 2.1 Animals

All experimental procedures were performed in accordance with institutional guidelines for animal studies and are approved by the Ethics Committee (US006 CREFRE ‐ CEEA‐122, 1710480320). Myeloid‐specific deletion of HMGB1 (HMGB1^ΔMac^) were generated crossing LysM‐CRE^+/−^ (a generous gift from a gift from Dr Pierre Gourdy, Toulouse France) with HMGB1^Flox ^mice (a generous gift from Dr Robert F. Schwabe, Columbia University, NY), littermates LysM‐CRE^−/−^ HMGB1^Flox/Flox ^(HMGB1^Flox^) were used as control. After randomization, 8 to 12‐week‐old male mice were used in this study. Mice were housed under specific pathogen‐free conditions at 20‐22°C and 50%‐60% humidity, with a 12 hour light/dark cycle and free access to water and food. For thermoneutrality studies, mice were housed at 30°C in a Noroit A‐Box (Noroit, Bouaye, France). Before running any experiments, mice were usually placed at 30°C for a 2‐week period of acclimation. At the time of sacrifice, tissues and organs were dissected, weighted and directly snap frozen in liquid nitrogen and stored at −80°C for gene expression and western blot analyses. For histological analysis, tissues were fixed in 10% formalin (Sigma‐Aldrich, St. Louis, MO, USA).

### 2.2 Genotyping

DNA extraction and polymerase chain reaction (PCR) were performed using Kapa mouse genotyping kit (Kapa Biosystems, Wilmington, MA) according to the manufacturer protocol. PCR reactions were performed using following primers: LysM‐CRE: 5′‐ACCGGTCGATGCAACGAGTGATGAG‐3′ (forward) and 5′‐AGTGCGTTCGAACGCTAGAGC‐3′ (reverse), LoxP1 5′‐TAAGAGCTGGGTAAACTTTAGGTG‐3′ (forward) and 5′‐GAAACAGACAAGCTTCAAACTGCT‐3′ (reverse), LoxP2 5′‐TGACAGGATACCCAGTGTTAGGGG‐3′ (forward) and 5′‐CCAGAGTTTAATCCACAGAAGAAA‐3′ (reverse). Deletion PCR was performed using LoxP1 forward and LoxP2 reverse primers.

### 2.3 Murine models of fibrosis

Liver chemically induced fibrosis was triggered by intraperitoneal injection (ip) of carbon tetrachloride (CCl_4_, 02671, Sigma‐Aldrich, St. Louis, MO, USA; 0.5 μL/g in corn oil, at a ratio of 1:3) for eight injections or by ip of thioacetamide (TAA, 163678, Sigma‐Aldrich, St. Louis, MO, USA) dissolved in NaCl 0.9% for 6 weeks (three injections per week) at increasing concentrations (first dose: 50 mg/kg, second dose: 100 mg/kg, third to sixth dose: 200 mg/kg, all following doses: 300 mg/kg) as previously described.^25^ Cholestatic‐induce liver fibrosis was induced in 8‐week‐old male mice by ligating the common bile duct for 21 days, as described.^26^ Kidney fibrosis was induced by unilateral ureteral obstruction (UUO) on 8‐week‐old mice. UUO was performed by ligating the ureter just below the renal pelvis for 7 days, as previously described.^27^ Transverse aortic constriction (TAC) was performed on 8 to 12‐week‐old male mice for 28 days. After anesthesia, the transverse aorta was isolated and TAC was performed by tying a waxed braided silk suture, to induce pressure overload and cardiac hypertrophy as previously described.^28^ For all surgical procedures, sham‐operated animals underwent the same operation except the suturing of inner organs.

### 2.4 Cardiac function assessment

The left ventricle dimensions were determined using echocardiography and (Time/Motion) mode acquisition from the parasternal short axis view at the level of the papillary muscles using a Vivid7 echograph and a 14 MHz transducer (i3L, GE Healthcare, Little Chalfont, UK). Images were transferred and analyzed offline with EchoPAC (GEHealthcare, Little Chalfont, UK).

### 2.5 Hepatic stellate cell isolation

Hepatic stellate cells were isolated from mice as described previously.^25^ Briefly, after cannulation of the inferior vena cava, the portal vein was cut, allowing retrograde step‐wise perfusion with pronase (Sigma‐Aldrich, St. Louis, MO, USA) and collagenase (Roche Diagnostics, Risch‐Rotkreuz, Switzerland) containing solutions, and subsequent 9.7% Nycodenz gradient centrifugation. Purity was assessed by vitamin A autofluorescence under a fluorescent microscope (Olympus 71IX). After 5 hours in dulbecco modified eagle medium (DMEM) 10% fetal calf serum (FCS), 1% Antibiotic‐Antimycotic (Gibco, Grand Island, NY, United States) and 0.1% Gentamicin (Gibco, Grand Island, NY, United States), stellate cells have been cultured with 1/1.74 diluted bone marrow‐derived macrophages (BMDM) conditioned media in DMEM 0.1% FBS for 24 hours.

### 2.6 Plasma analysis

Aspartate transaminase (AST) and alanine transaminase (ALT) levels were determined in plasma by the Phenotypage‐CREFRE facility using a Pentra400 biochemical analyzer (HORIBA Medical, Kyoto, Japan). HMGB1 circulating levels were assessed by ELISA (ST51011; IBL International, Hamburg, Germany) according to the manufacturer guidelines.

### 2.7 Bone marrow‐derived macrophages

Bone marrow cells were obtained by flushing the cavity of femurs and tibia of HMGB1^Flox^ and HMGB1^ΔMac^ mice (8 week old) in sterile DMEM F12 supplemented with 10% FCS and 1% penicillin/streptomycin (Sigma‐Aldrich, St. Louis, MO, USA). Cells were filtered on a 42 μm cell‐strainer, and after erythrocyte lysis, harvested cells are plated and treated with 10 ng/mL recombinant murine macrophage colony stimulating factor (M‐CSF) (PeproTech, Inc, Rocky Hill, NJ, USA) for 7 days. The medium was renewed every 2 days with DMEM 10% FCS with 10 ng/mL M‐CSF. Then, BMDM were treated with 10 ng/mL of LPS (055:B5, Sigma‐Aldrich, St. Louis, MO, USA) for 1 hour and 30 minutes, then washed two times to remove LPS using phosphate buffered saline (PBS), and conditioned media were generated in DMEM 0.1% FCS during 48 hours.

### 2.8 Immunocytofluorescence

Bone marrow‐derived macrophages were fixed after 7 days of differentiation, with 10% formalin. Cells were permeabilized with 0.1% Triton and stained with a primary antibody against HMGB1 (1:200, ab18256; Abcam, Cambridge, UK) and Alexa Fluor 546‐conjugated secondary antibody (1:800, A11010; Life Technologies, Carlsbad, CA, USA). Nuclei were stained with DAPI (Sigma‐Aldrich, St. Louis, MO, USA). Confocal microscopy was performed on an LSM 780 confocal laser microscope (Zeiss, Oberkochen, Germany) using a 63× oil immersion lens.

### 2.9 Stromal vascular fraction preparation

Mice were anesthetized with pentobarbital (50 mg/kg), then perfused with PBS to wash out blood from tissues. Mice were euthanized and tissues were harvested. Liver and lungs were digested with collagenase D (2.5 mg/mL; Roche Diagnostics, Risch‐Rotkreuz, Switzerland) under agitation for 20 minutes at 37°C. After centrifugation at 600 g for 10 minutes, stromal cells were separated in 30% percoll (GE Healthcare, 17‐0891‐01, Upsale, Sweden). After centrifugation at 600 *g* for 15 minutes, the pellet containing stromal cells were incubated with erythrocyte lysis buffer for 10 minutes followed by another centrifugation (600 *g*, 10 minutes) and resuspension in PBS.

### 2.10 Western blotting

Tissues were homogenized in RIPA buffer (TRIS 20 mmol/L, NaCl 150 mmol/L, EDTA 1 mmol/L, EGTA 1 mmol/L, TRITON X100 1%, Tetra‐Sodium Pyrophosphate 2.5 mmol/L, B‐Glycerophosphate 1 mmol/L, Sodium orthovanadate 1 mmol/L) containing proteases and phosphatases inhibitors (Sigma‐Aldrich, St. Louis, MO, USA) using Precellys sample lyzer (Bertin Technologies, Montigny le Bretonneux, France). Western blots were performed using standard procedures using antibodies against HMGB1 (1:1000, ab18256; Abcam, Cambridge, UK) and α‐smooth muscle actin (α‐SMA) (1:1000, ab5694; Abcam, Cambridge, UK). GAPDH (1:2000, ab181602; Abcam, Cambridge, UK), was used as a loading control.

### 2.11 Gene expression

RNA was extracted using GenJET RNA purification kit (ThermoScientific, Waltham, MA, USA) and DNAse treatment (Qiagen, Hilden, Germany). After dosage with Xpose (Trinean, Gentbrugge, Belgium), reverse transcription was performed using High Capacity cDNA reverse transcription kit (Applied Biosystems, Foster City, CA, USA) according to the manufacturer protocol. Real‐Time‐qPCR (RT‐qPCR) was performed with indicated primer pairs gene expression is normalized using *Rplp0 *reference gene expression. Primer sequences were as follows: *Acta2*, 5′‐GTCCCAGACATCAGGGAGTAA‐3′ (forward) and 5′‐TCGGATACTTCAGCGTCAGGA‐3′ (reverse), *Col1a1*, 5′‐TGTGTGCGATGACGTGCAAT‐3′ (forward) and 5′‐GGGTCCCTCGACTCCTACA‐3′ (reverse), *Col3a1*, 5′‐AAGGCGAATTCAAGGCTGAA‐3′ (forward) and 5′‐TGTGTTTAGTACAGCCATCCTCTAGAA‐3′ (reverse), *Emr1*, 5′‐TGACAACCAGACGGCTTGTG‐3′ (forward) and 5′‐GCAGGCGAGGAAAAGATAGTGT‐3′ (reverse), *Rplp0*, 5′‐AGTCGGAGGAATCAGATGACGAT‐3′ (forward) and 5′‐GGCTGACTTGGTTGCTTTGG‐3′ (reverse), *Tnfα *5′‐TGGGACAGTGACCTGGACTGT‐3′ (forward) and 5′‐TTCGGAAAGCCCATTTGAGT‐3′ (reverse), *Il6*, 5′‐GCCCACCAAGAACGATAGTCA‐3′ (forward) and 5′‐CAAGAAGGCAACTGGATGGAA‐3′ (reverse)*, Il1β*, 5′‐CAACCAACAAGTGATATTCTCGATG‐3′ (forward) and 5′‐GATCCACACTCTCCAGCTGCA‐3′ (reverse), *Ccn2*, 5′‐GGCATCTCCACCCGAGTTAC‐3′ (forward) and 5′‐GATTTTAGGTGTCCGGATGCA‐3′ (reverse). For *Col1a1,* *Col1a2, and Acta2 *mRNA expression displayed in Figure S4, RNA were extracted using High Pure RNA Isolation kit (Roche Diagnostics, Risch‐Rotkreuz, Switzerland) and qPCR analysis was conducted using Taqman‐primer probes (Applied Biosystems, Foster City, CA, USA).

### 2.12 Histology and immunohistochemistry

10% formalin‐fixed samples were paraffin embedded and sliced at 5 µm. For picrosirius red staining, sections were dipped for 1.5 hours in picric acid containing 1% direct red 80 (365548; Sigma‐Aldrich, St. Louis, MO, USA) and 0.5% fast green (F7258; Sigma‐Aldrich, St. Louis, MO, USA). Immunohistochemistry was performed using anti‐mouse α‐SMA (ab5694; Abcam, Cambridge, UK) followed by a biotinylated goat anti‐rat antibody (BA‐9400; Vector Laboratories, Inc, Burlingame, CA) and streptavidin‐HRP (DY998; R&D systems, Minneapolis, MN) with 3‐3'‐Diaminobenzidine (DAB) as substrate (11718096001; Sigma‐Aldrich, St. Louis, MO, USA). Stained slides were scanned using a Nanozoomer scanner (Hamamatsu Photonics, Hamamatsu City, Japan). Quantification was performed using Photoshop (Adobe Systems, San Jose, CA, USA) software.

### 2.13 Statistics

Analyses are performed using GraphPad Prism 7 (GraphPad Software, La Jolla, CA, USA). All data are expressed as mean ± SEM, except otherwise indicated, statistical significance was determined by *t* test, Mann‐Whitney or two‐way ANOVA tests. *P* values <0.05 were considered significant (*P < 0.05; **P < 0.01; ***P < 0.001; ****P < 0.0001).

## 3 RESULTS

### 3.1 Liver HMGB1 levels are upregulated during liver fibrosis

**Figure 1:**
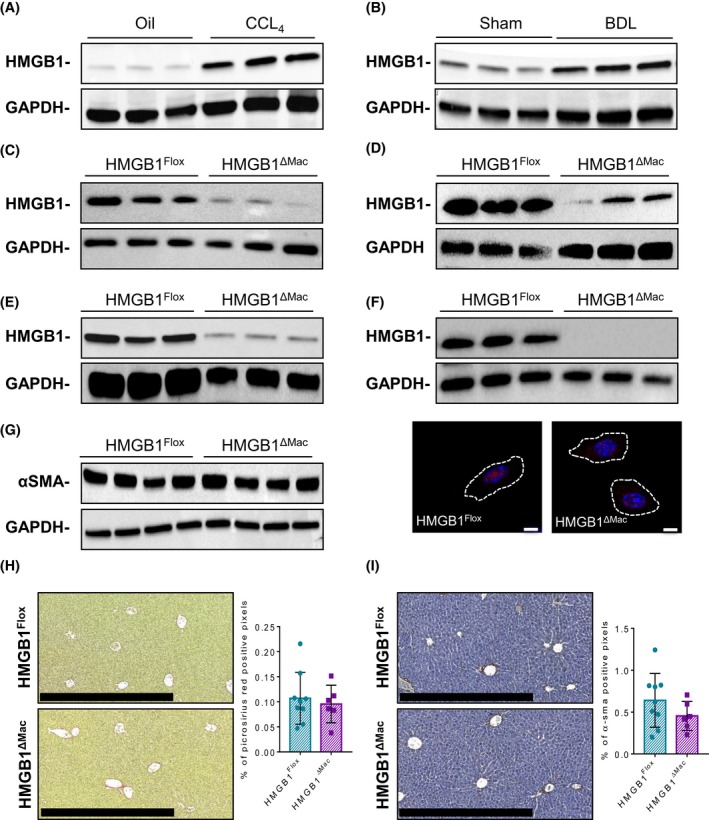
High mobility group B1 (HMGB1) regulation during fibrosis and validation and characterization of macrophage‐specific deletion of HMGB1. Representative immunoblotting against HMGB1 in oil (n = 9) or CCL_4_‐treated (n = 15) liver using glyceraldehyde‐3‐phosphate dehydrogenase (GAPDH) protein as a loading control (A). Representative immunoblotting against HMGB1 in liver extracts from sham (n = 9) or BDL (n = 4) animals using GAPDH protein as a loading control (B). Peritoneal macrophages isolated from HMGB1^Flox^ (n = 3) and HMGB1^ΔMac ^mice (n = 3) were analyzed by immunoblotting against HMGB1 and GAPDH (C). Cells from liver stromal vascular fraction, isolated from HMGB1^ΔMac ^and HMGB1^Flox^ mice, were analyzed by immunoblotting against HMGB1 and GAPDH (D). Cells from lung stromal vascular fraction, isolated from HMGB1^Flox^ (n = 3) and HMGB1^ΔMac ^mice (n = 3), were analyzed by immunoblotting against HMGB1 and GAPDH (E). Bone marrow‐derived macrophages (BMDM) isolated from HMGB1^Flox^ (n = 3) and HMGB1^ΔMac ^mice (n = 3) were analyzed by immunoblotting against HMGB1 and GAPDH (higher panel). BMDM isolated from HMGB1^Flox^ and HMGB1^ΔMac ^mice were analyzed by immunofluorescence confocal microscopy to detect HMGB1 (red) while nuclei were stained with DAPI (blue) (lower panel). Scale bar: 5 μm (F). Liver extracts of HMGB1^Flox^ (n = 9) and HMGB1^ΔMac^ (n = 6) mice were analyzed by western blotting directed against α‐smooth muscle actin (α‐SMA) (G). Representative images of liver fibrosis determined by picosirius Red staining from HMGB1^Flox^ (n = 9) and HMGB1^ΔMac^ (n = 6) mice. Scale bar: 500 μm (H). Representative images of myofibroblast activation determined by α‐SMA immunostaining from HMGB1^Flox^ (n = 9) and HMGB1^ΔMac^ (n = 6) mice liver sections. Scale bar: 500 μm (I)

As HMGB1 is thought to be a determinant player in fibrogenesis, we first characterized the expression levels of HMGB1 during fibrosis progression in the liver. Using two models of chemically (CCL_4_) or surgically (BDL)‐induced liver fibrosis, we show that HMGB1 protein levels were strongly upregulated in fibrotic livers compared to control livers (Figure 1A,B) suggesting a possible role of HMGB1 during fibrogenesis. To determine whether macrophage‐derived HMGB1 could be responsible for fibrosis progression, we generated mice specifically deleted for *Hmgb1* gene in macrophages (HMGB1^ΔMac^) by crossing LysM‐Cre^29^ and HMGB1‐floxed transgenic mice.^30^ Besides getting a clear recombination in the *Hmgb1* gene locus (Figure S1A,B), western blotting analysis demonstrated an efficient deletion of HMGB1, in vivo in naïve peritoneum macrophages or in resident macrophages from stromal vascular fraction of liver or lungs with 88%, 55%, and 98% of deletion, respectively (Figure 1C‐E) and in vitro in BMDM with 99% deletion using immunoblotting or immunocytofluorescence (Figure 1F). Upon basal conditions, compared to HMGB1^Flox^, HMGB1^ΔMac^ mice did not show any detectable differences of fibrosis features with comparable protein levels of α‐SMA protein as measured by immunoblotting (Figure 1G) and normal picrosirius red staining surrounding liver vessels and staining of vascular smooth muscle cells (Figure 1H,I).

### 3.2 HMGB1^ΔMac^ mice do not have an increased liver fibrogenesis after CCL_4_ treatment

**Figure 2:**
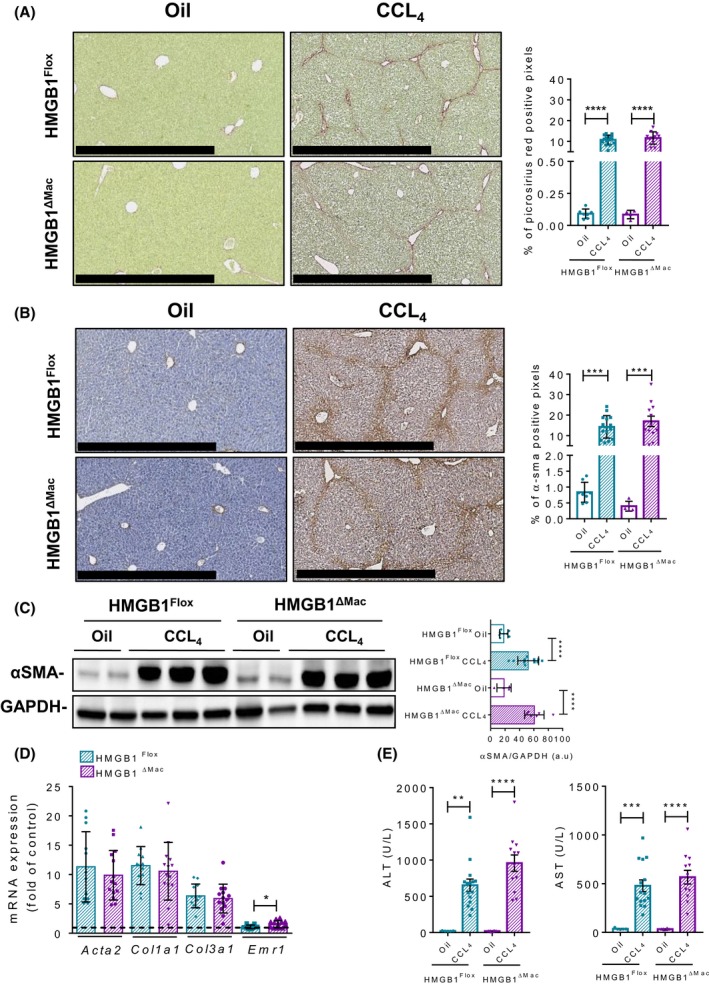
Macrophage‐specific deletion of high mobility group B1 (HMGB1) does not modify liver fibrogenesis after CCL_4_ treatment. Representative pictures of picrosirius red staining of HMGB1^Flox^ mice and HMGB1^ΔMac^ of liver section and quantification of positive pixels per liver section. Scale bar: 500 μm (A). Representative pictures of immunohistochemical staining with an antibody against α‐smooth muscle actin (α‐SMA) and quantification of positive pixels per liver section. Scale bar: 500 μm (B). Liver extracts from HMGB1^Flox^ mice and HMGB1^ΔMac^ were analyzed by western blotting directed against α‐SMA (C). Liver mRNA expression levels of classical fibrosis markers were detected using real‐time RT‐PCR, the dotted line indicates the baseline (D). Hepatic injury in HMGB1^Flox^ mice and HMGB1^ΔMac^ was determined by alanine transaminase (ALT) (left panel) and aspartate transaminase (AST) (right panel) levels (E). Statistical analysis was performed using Mann‐Whitney test. Data are expressed as means ± SEM. n = 9 in HMGB1^Flox^‐oil group; n = 6 in HMGB1^ΔMac^‐oil group; n = 15 in HMGB1^Flox^‐CCL_4_ group; n = 12 in HMGB1^ΔMac^‐CCL_4_ group. *P < 0.05, **P < 0.01, ***P < 0.001, ****P < 0.0001 vs oil/CCL_4_

To determine whether macrophage‐derived HMGB1 is playing a key role in fibrogenesis, we first studied liver fibrosis. In this purpose, HMGB1^Flox^ and HMGB1^ΔMac^ mice were treated with eight i.p injections of carbon tetrachloride (CCL_4_) and were euthanized 2 days after the last injection. *Hmgb1* gene deletion has been carefully monitored using deletion PCR on whole liver DNA extract from both HMGB1^Flox^ and HMGB1^ΔMac ^mice (Figure S1C). Following CCL_4 _treatment, HMGB1^Flox^ mice exhibited a significant accumulation of collagen compared to oil‐treated mice as shown by picrosirius red staining (Figure 2A), but no differences were detected between HMGB1^Flox^ and HMGB1^ΔMac^ mice. Similarly, HMGB1^Flox^ and HMGB1^ΔMac ^mice displayed comparable levels of α‐SMA immunostaining (Figure 2B). Moreover, there were no differences in α‐SMA protein levels (Figure 2C) as well as similar mRNA expression levels of fibrogenic and pro‐inflammatory markers such as *Acta2*, *Ccn2*, *Col1a1*, *Col3a1, Tnfα*, and *Il‐6* except for *Emr1* and *Il‐1β* which were upregulated in HMGB1^ΔMac ^mice (Figure 2D; Figure S2A). Finally, while CCL_4_ treatment induced a pronounced liver injury compared to oil‐treated mice, there were no differences in serum ALT or AST levels between HMGB1^Flox^ and HMGB1^ΔMac ^mice (Figure 2E). To rule out a possible overpowering effect of 8× CCL_4 _on HMGB1 potential fibrogenic effect, we used a less severe experimental setup, where HMGB1^Flox^ and HMGB1^ΔMac ^mice were subjected to only four injections of CCL_4_. Even on a shorter CCL_4_ model, HMGB1^Flox^ and HMGB1^ΔMac^ mice displayed the same extent of fibrosis in the liver (Figures S2F & S3A‐E), suggesting that macrophage‐derived HMGB1 does neither contribute to early fibrogenic events during fibrosis progression.

### 3.3 HMGB1^ΔMac^ mice do not display increased TAA‐induced liver fibrosis

**Figure 3:**
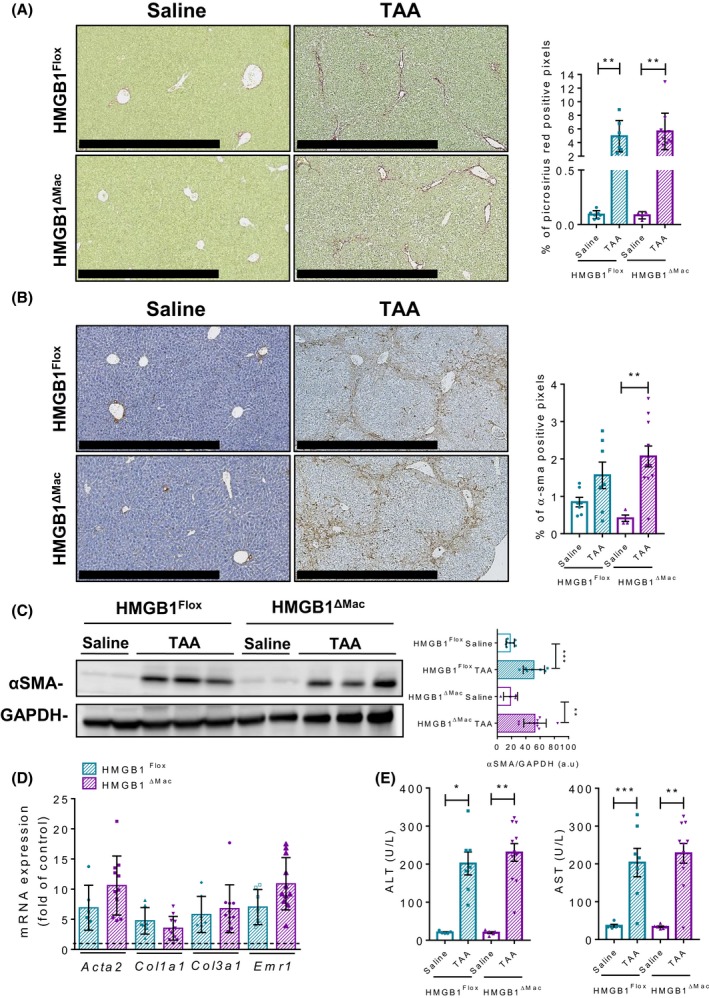
Macrophage‐specific deletion of high mobility group B1 (HMGB1) does not modify liver fibrogenesis after TAA treatment. Representative pictures of picrosirius red staining of HMGB1^Flox^ mice and HMGB1^ΔMac^ of liver section and quantification of positive pixels per liver section. Scale bar: 500 μm (A). Representative pictures of immunohistochemical staining with an antibody against α‐smooth muscle actin (α‐SMA) and quantification of positive pixels per liver section. Scale bar: 500 μm (B). Liver extracts from HMGB1^Flox^ mice and HMGB1^ΔMac^ were analyzed by western blotting directed against α‐SMA (C). Liver mRNA expression levels of classical fibrosis markers were detected using real‐time RT‐PCR, the dotted line indicates the baseline (D). Hepatic injury in HMGB1^Flox^ mice and HMGB1^ΔMac^ was determined by alanine transaminase (ALT) (left panel) and aspartate transaminase (AST) (right panel) levels (E). Statistical analysis was performed with Mann‐Whitney test. Data are expressed as means ± SEM. n = 6 in HMGB1^Flox^‐saline group; n = 6 in HMGB1^ΔMac^‐saline group; n = 7 in HMGB1^Flox^‐TAA group; n = 11 in HMGB1^ΔMac^‐TAA group. *P < 0.05, **P < 0.01, ***P < 0.001 vs saline/TAA

To confirm these findings in a second well‐established model of chemical‐induced liver fibrogenesis, we next challenged mice using TAA. HMGB1^ΔMac ^and HMGB1^Flox^ mice were treated with 18 i.p injections of TAA. As performed on CCL_4_ model, *Hmgb1* gene deletion was carefully monitored using deletion PCR on whole liver DNA extract from both HMGB1^Flox^ and HMGB1^ΔMac^ mice (Figure S1D). Similar to our results obtained in the CCL_4_ model, we did not detect any differences between HMGB1^Flox^ and HMGB1^ΔMac ^mice. After TAA treatment, HMGB1^Flox^ mice exhibited a pronounced deposition of fibrillar collagen and α‐SMA staining compared to saline‐treated mice but the increase in fibrotic markers was identical between HMGB1^Flox^ and HMGB1^ΔMac ^mice (Figure 3A,B). In addition, α‐SMA (Figure 3C) protein levels, as well as mRNA expression of classical fibrosis and inflammatory markers such as *Acta2*, *Ccn2, Col1a1*, *Col3a1*, *Emr1, *and *Il‐6* except for *Tnfα*, *Il‐1β* which were upregulated in HMGB1^ΔMac ^mice (Figure 3D; Figure S2B) and serum levels of liver injury markers (Figure 3E), were comparable in HMGB1^Flox^ and HMGB1^ΔMac ^mice subjected to TAA treatment.

### 3.4 HMGB1^ΔMac^ mice do not have an increased hepatic fibrogenesis after BDL

**Figure 4:**
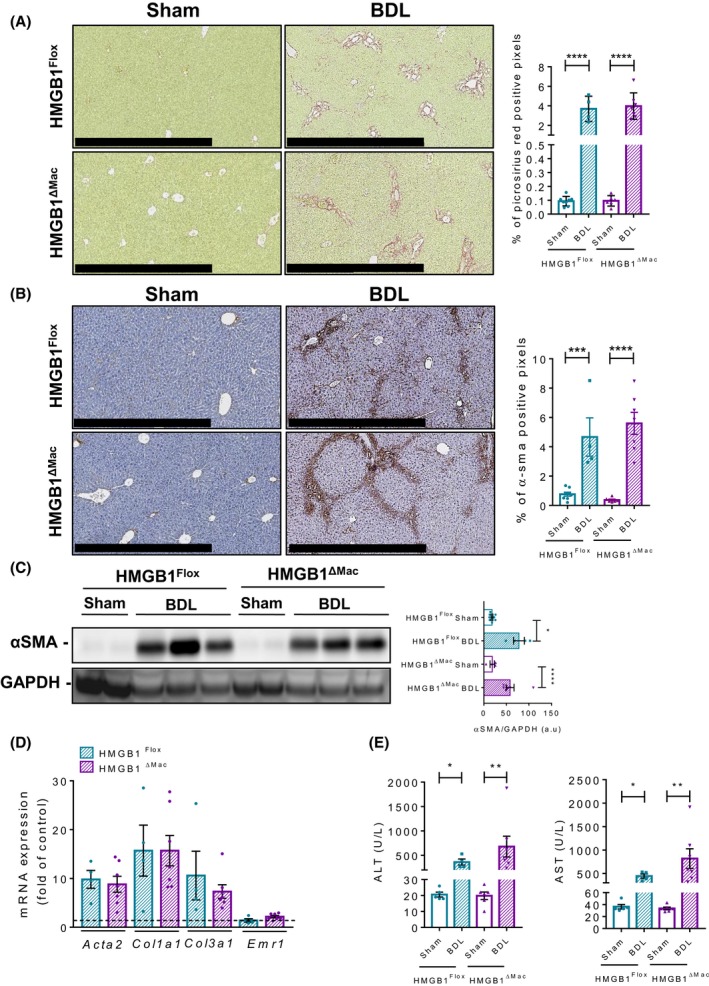
Macrophage‐specific deletion of high mobility group B1 (HMGB1) does not alter liver fibrogenesis after BDL. Representative pictures of picrosirius red staining of HMGB1^Flox^ mice and HMGB1^ΔMac^ of liver section and quantification of positive pixels per liver section. Scale bar: 500 μm (A). Representative pictures of immunohistochemical staining with an antibody against α‐smooth muscle actin (α‐SMA) and quantification of positive pixels per liver section. Scale bar: 500 μm (B). Liver extracts from HMGB1^Flox^ mice and HMGB1^ΔMac^ were analyzed by western blotting directed against α‐SMA (C). Liver mRNA expression levels of classical fibrosis markers were detected using real‐time RT‐PCR, the dotted line indicates the baseline (D). Hepatic injury in HMGB1^Flox^ mice and HMGB1^ΔMac^ was determined by alanine transaminase (ALT) (left panel) and aspartate transaminase (AST) (right panel) levels (E). Statistical analysis was performed with Mann‐Whitney test. Data are expressed as means ± SEM. n = 6 in HMGB1^Flox^‐sham group; n = 6 in HMGB1^ΔMac^‐sham group; n = 4 in HMGB1^Flox^‐BDL group; n = 7 in HMGB1^ΔMac^‐BDL group. *P < 0.05, **P < 0.01, ***P < 0.001, ****P < 0.0001 vs sham/BDL

To corroborate these findings, we used a complementary model of cholestatic liver injury, bile duct ligation (BDL). As done previously, *Hmgb1* gene deletion was carefully monitored using deletion PCR on whole liver DNA extracted from both HMGB1^Flox^ and HMGB1^ΔMac^ mice (Figure S1E). Twenty‐one days after BDL, HMGB1^Flox ^mice displayed severe hepatic fibrosis as shown by picrosirius red staining but again no differences were detected between HMGB1^Flox^ and HMGB1^ΔMac^ mice (Figure 4A). Expression of α‐SMA was also increased in HMGB1^Flox^ mice assessed by immunohistochemistry and immunoblotting but with no differences compared to HMGB1^ΔMac^ mice (Figure 4B,C). mRNA expression of fibrogenic markers *Acta2*, *Ccn2, Col1a1*, *Col3a1*, *Acta2*, *Col1a1*, *Col3a1,* and inflammatory markers *Emr1 *and *Il‐6,* except for *Tnfα*, *Il‐1β* which were upregulated in HMGB1^ΔMac ^mice (Figure 4D; Figure S2C) as well as serum ALT and AST levels, were also similar in HMGB1^Flox^ and HMGB1^ΔMac^ mice (Figure 4D,E).

As several studies suggested a direct effect of HMGB1 on hepatic stellate cells (HSCs) in the development of liver fibrosis,^31^, ^32^ we sought to further study the role macrophage derived‐HMGB1. Because (a) macrophages have a key role in promoting HSC activation, HSC survival, and liver fibrosis,^26^, ^33^ (b) LPS promotes liver fibrosis^34^ and (c) macrophages can release HMGB1 following LPS stimulation, we used LPS‐stimulated conditioned media from HMGB1^Flox^ and HMGB1^ΔMac^ macrophages to determine the role of macrophage‐derived HMGB1 in HSC activation. Of note, LPS strongly increased HMGB1 release over time, with a peak at 48 hours, which was almost completely blocked in HMGB1‐deleted macrophages (Figure S4A). Forty‐eight hour‐conditioned medium (CM) from LPS‐stimulated BMDM increased the activation of HSC compared to CM from unstimulated BMDM as shown by mRNA expression fibrogenic markers *Col1a1*, *Col1a2,* and *Acta2* (Figure S4B‐D). However, HSC activation did not differ between CM prepared from bone marrow isolated from HMGB1^Flox^ and HMGB1^ΔMac^ mice, demonstrating that the pro‐fibrotic effects of BMDM are independent of HMGB1 (Figure S4B‐D).

### 3.5 Macrophage‐derived HMGB1 does not play a crucial role in UUO‐induced kidney fibrosis

**Figure 5:**
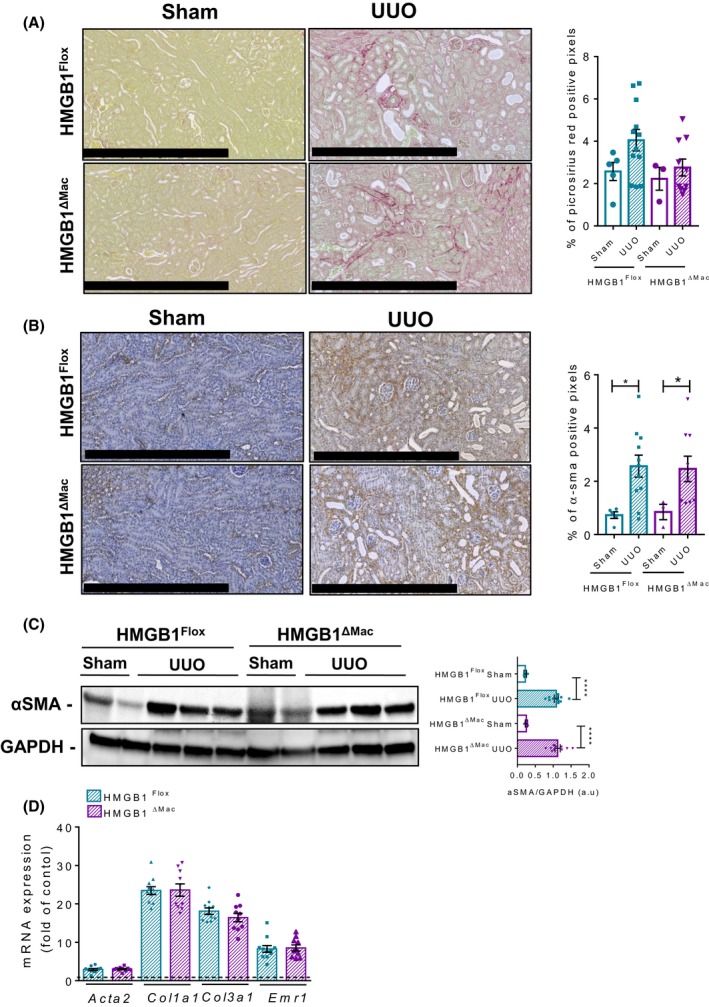
Macrophage‐specific deletion of high mobility group B1 (HMGB1) does not play a role in UUO‐induced kidney fibrosis. Representative pictures of picrosirius red staining of HMGB1^Flox^ mice and HMGB1^ΔMac^ of kidney section and quantification of positive pixels per kidney section. Scale bar: 500 μm (A). Representative pictures of immunohistochemical staining with an antibody against α‐smooth muscle actin (α‐SMA) and quantification of positive pixels per kidney section. Scale bar: 500 μm (B). Kidney extracts from HMGB1^Flox^ and HMGB1^ΔMac^ mice were analyzed by western blotting directed against α‐SMA (C). Kidney mRNA expression levels of classical fibrosis markers were detected using real‐time RT‐PCR, the dotted line indicates the baseline (D). Statistical analysis was performed with Mann‐Whitney test. Data are expressed as means ± SEM. n = 3 in HMGB1^Flox^‐sham group; n = 3 in HMGB1^ΔMac^‐sham group; n = 12 in HMGB1^Flox^‐UUO group; n = 10 in HMGB1^ΔMac^‐UUO group. *P < 0.05, **P < 0.01, ***P < 0.001 vs sham/UUO

As the results collected on three relevant injury models unexpectedly showed no effects of HMGB1 deletion on liver fibrosis, we next tested whether the role of macrophage HMGB1 in kidney fibrosis, where fibrotic mechanisms are slightly different compared to the liver. For this purpose, we employed a classical model of tubulointerstitial fibrosis induced by UUO.^27^ As performed in the liver, *Hmgb1* gene deletion was carefully monitored using deletion PCR on whole kidney DNA extract from both HMGB1^Flox^ and HMGB1^ΔMac^ mice (Figure S1F). Seven days after UUO, HMGB1^Flox ^mice displayed typical fibrosis features with a drastic deposition of fibrillar collagen and myofibroblasts activation (Figure 5A,B) compared to sham‐operated mice, as determined by immunohistochemistry. But similar to the liver fibrogenesis models, no differences were detected between HMGB1^Flox^ and HMGB1^ΔMac^ mice in UUO‐induced kidney fibrosis progression. Moreover, α‐SMA protein levels (Figure 5C), as well as mRNA expression of fibrogenic and inflammatory genes *Acta2*, *Ccn2*, *Col1a1*, *Col3a1*, *Emr1, Il‐1β, *and *Il‐6* were comparable between HMGB1^Flox^ and HMGB1^ΔMac ^mice in the harvested kidney following UUO, except for *Tnfα* which was upregulated in HMGB1^ΔMac ^mice (Figure 5D; Figure S2D).

### 3.6 HMGB1^ΔMac^ mice do not display an increased cardiac fibrosis after TAC

**Figure 6:**
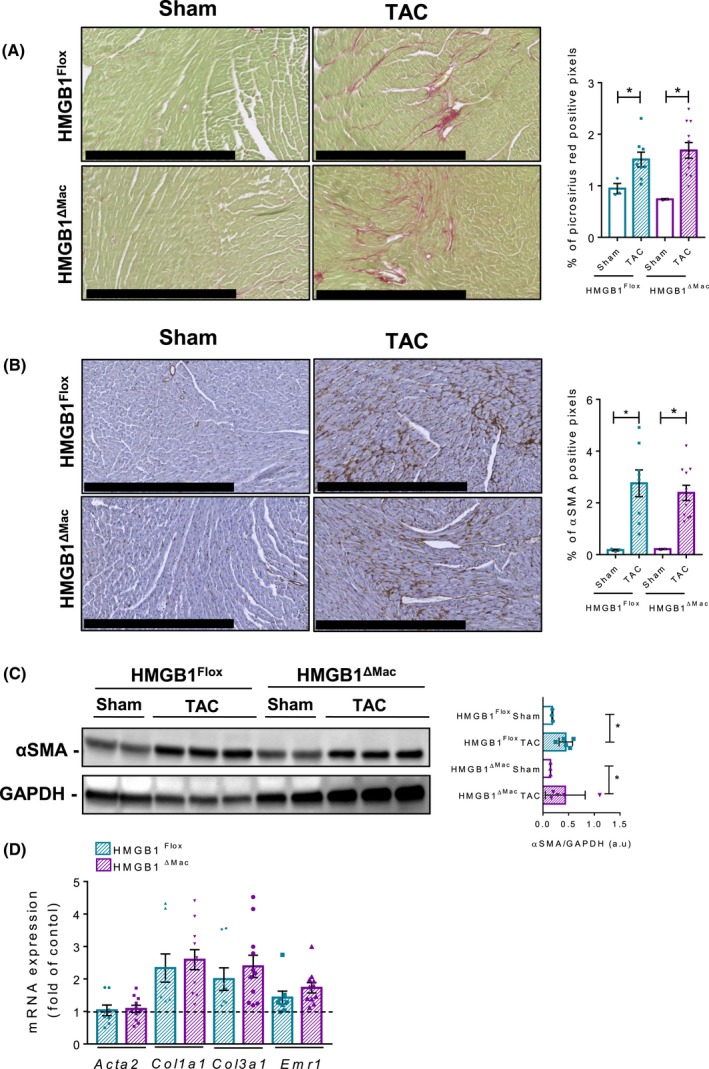
Macrophage‐specific deletion of high mobility group B1 (HMGB1) does not modify cardiac fibrogenesis after transverse aortic constriction (TAC). Representative pictures of picrosirius red staining of HMGB1^Flox^ mice and HMGB1^ΔMac^ of heart section and quantification of positive pixels per heart section. Scale bar: 500 μm (A). Representative pictures of immunohistochemical staining with an antibody against α‐smooth muscle actin (α‐SMA) and quantification of positive pixels per heart section. Scale bar: 500 μm (B). Heart extracts from HMGB1^Flox^ mice and HMGB1^ΔMac^ were analyzed by western blotting directed against α‐SMA (C). Heart mRNA expression levels of classical fibrosis markers were detected using real‐time RT‐PCR, the dotted line indicates the baseline (D). Statistical analysis was performed with Mann‐Whitney test. Data are expressed as means ± SEM. n = 3 in HMGB1^Flox^‐sham group; n = 3 in HMGB1^ΔMac^‐sham group; n = 8 in HMGB1^Flox^‐TAC group; n = 11 in HMGB1^ΔMac^‐TAC group. *P < 0.05, **P < 0.01, ***P < 0.001 vs sham/TAC

In addition to the liver and kidney, we finally assessed the impact of HMGB1 deletion in macrophages on fibrosis progression in the heart as it has been reported recently that HMGB1 could play a key role in cardiac fibrosis,^22^ using TAC as a well‐established and clinically relevant model of cardiac fibrosis.^35^ As performed previously, *Hmgb1* gene deletion was carefully monitored using deletion PCR on whole heart DNA extract from both HMGB1^Flox^ and HMGB1^ΔMac^ mice (Figure S1G). Twenty‐eight days after TAC, HMGB1^Flox ^mice displayed pronounced impairments of cardiac function such as left ventricular end‐systolic and ‐diastolic dimension (Table1) compared to sham‐operated animals, accompanied with a strong collagen deposition (Figure 6A). But once again, HMGB1^Flox^ and HMGB1^ΔMac^ mice subjected to TAC showed a similar extent of functional alterations and extracellular matrix accumulation determined by picrosirius red staining or α‐SMA immunohistochemistry and immunoblotting (Table 1; Figure 6A‐C). In addition, similar levels of mRNA expression of classical fibrogenic markers such as *Acta2*, *Ccn2*, *Col1a1*, and *Col3a1 mRNA*, and inflammatory marker *Emr1, Tnfα,* and *Il‐1b* (except for *Il‐6* which was upregulated in HMGB1^ΔMac ^mice) mRNA were detected using real‐time qPCR in operated HMGB1^Flox^ and HMGB1^ΔMac ^mice (Figure 6D; Figure S2E).

**Table 1:** Echocardiographic and anatomical analysis as a function of transverse aortic constriction (TAC). Statistical analysis was performed with a two‐way ANOVA analysis

Time post surgery	4 wk			
Genotype	HMGB1^Flox^ Sham	HMGB1^ΔMac^ Sham	HMGB1^Flox^ + TAC	HMGB1^ΔMac^ + TAC
Echocardiographic parameters				
LVEDD (mm)	3.443 ± 0.12	3.435 ± 0.11	4.018 ± 0.15**	3.846 ± 0.12**
LVESD (mm)	1.955 ± 0.09	1.98 ± 0.12	2.604 ± 0.22*	2.39 ± 0.16*
FS (%)	43.25 ± 1.93	42.25 ± 1.79	36.25 ± 3.29	38.36 ± 2.37
Anatomical parameters				
BW (g)	31.94 ± 0.54	30.49 ± 1.25	27.29 ± 0.90**	28.73 ± 0.69**
Heart weight (mg)	144.3 ± 7.14	152 ± 3.51	169.5 ± 11.21*	174.3 ± 6.38*
HW/TL (mg/mm)	78.27 ± 3.81	82.51 ± 1.70	97.8 ± 6.22**	98.6 ± 3.64**

Data are expressed as means ± SEM. n = 4 in HMGB1^Flox^/sham group; n = 4 in HMGB1^ΔMac^/sham group; n = 8 in HMGB1^Flox^/TAC group; n = 13 in HMGB1^ΔMac^/TAC group.

BW, body weight; FS, fractional shortening; HMGB1, high mobility group B1; HW/TL, heart weight to tíbia length ratio; LVEDD, left ventricular end‐diastolic dimension; LVESD, left ventricular end‐systolic dimension; TAC, transverse aortic constriction.

* *P* < 0.05.

** *P* < 0.01 vs sham/TAC.

### 3.7 Thermoneutral housing has no incidence on fibrosis progression in HMGB1^ΔMac^ mice compared to HMGB1^Flox ^mice

**Figure 7:**
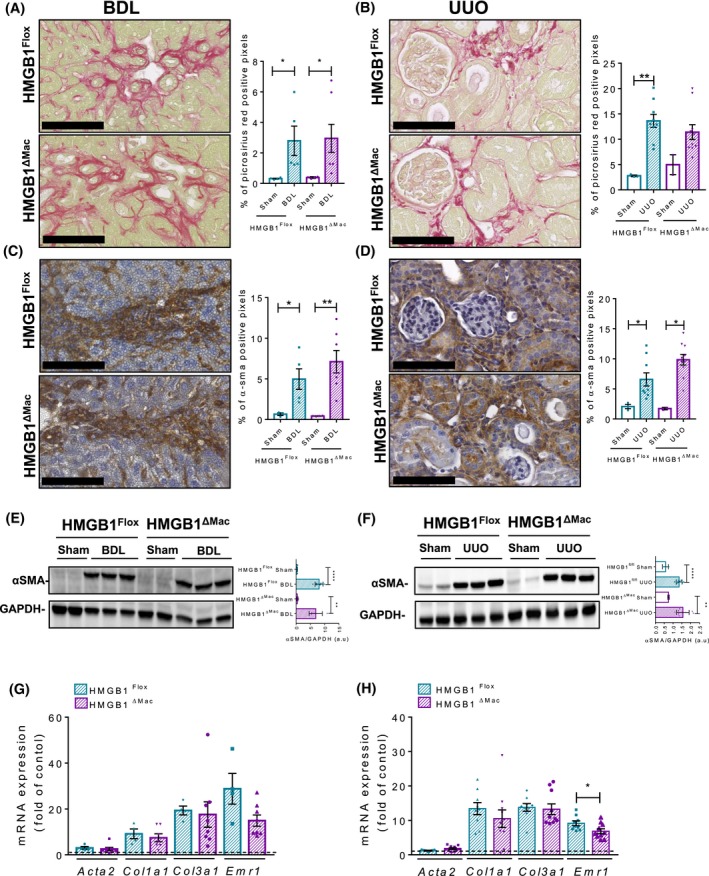
Thermoneutral housing has no incidence of fibrosis progression in mice with macrophage‐specific HMGB1 deletion (HMGB1^ΔMac^) compared to HMGB1^Flox ^mice. Representative pictures of picrosirius red staining of sham and operated HMGB1^Flox^ mice and HMGB1^ΔMac^ of liver (A) and kidney (B) and quantification of positive pixels per tissue section. Scale bar: 100 μm (A,B). Representative pictures of immunohistochemical staining of sham and operated HMGB1^Flox^ and HMGB1^ΔMac^ mice with an antibody against α‐smooth muscle actin (α‐SMA) in the liver (C) and kidney (D) and quantification of positive pixels per tissue section. Scale bar: 100 μm (C,D). Liver (E) and kidney (F) extracts from sham and operated HMGB1^Flox^ and HMGB1^ΔMac^ mice were analyzed by western blotting directed against α‐SMA (E,F). Liver (G) and kidney (H) mRNA expression levels of classical fibrosis markers expressed in fold of sham‐operated animals (dotted line) were detected using real‐time RT‐PCR, the dotted line indicates the baseline (G,H). Statistical analysis was performed with Mann‐Whitney test. Data are expressed as means ± SEM. n = 4 (liver) n = 3 (kidney) in HMGB1^Flox^‐sham group; n = 3 (liver & kidney) in HMGB1^ΔMac^‐sham group; n = 5 in HMGB1^Flox^‐bile duct ligation (BDL) group; n = 7 in HMGB1^ΔMac^‐BDL group; n = 12 in HMGB1^Flox^‐unilateral ureteral obstruction (UUO) group; n = 12 in HMGB1^ΔMac^‐UUO group. *P < 0.05, **P < 0.01, ***P < 0.001, ****P < 0.0001 vs sham/BDL or sham/UUO

In the recent years, several striking studies have demonstrated that for rodent models, as opposed to thermoneutral housing (30‐32°C), room temperature housing (20‐22°C) initiates and maintains a chronic cold stress that dramatically impairs physiology and immune response (eg, to inflammatory stimulus).^36^, ^37^, ^38^ As a severe alteration of immune responses has pronounced consequences on fibrosis progression,^39^, ^40^ we hypothesized that thermoneutral housing, rather than room temperature housing performed so far, might be a more relevant context to evaluate the impact of macrophage‐derived HMGB1 deletion on fibrosis development. For this purpose, after 2 weeks of acclimation at 30°C, HMGB1^Flox^ and HMGB1^ΔMac^ mice were subjected to two fibrosis models: BDL‐induced liver fibrosis and UUO‐induced kidney fibrosis. As done at room temperature conditions*, Hmgb1* gene deletion has been carefully monitored using deletion PCR on whole liver or kidney DNA extract from both HMGB1^Flox^ and HMGB1^ΔMac ^mice (Figure 1H‐J). As expected, we found that compared to room temperature, thermoneutral housing affected global mouse physiology, and remarkably HMGB1 circulating levels were higher in mice housed at 30°C compare to 20°C housing conditions (Figure 5A). In addition, serum ALT and AST levels (Figure 5B‐C) and liver inflammation and fibrosis (Figure 5D‐E) were increased when mice are housed at 30°C compared to 20°C suggesting a higher hepatocyte turnover at 30°C. In both models and similar to what we observed at room temperature, HMGB1^Flox ^mice exhibited a severe deposition of collagen and myofibroblast activation compared to sham‐operated animals (Figure 7A‐D). But similar to room temperature housing, HMGB1^Flox^, and HMGB1^ΔMac^ mice displayed the same extent of fibrosis progression induced by either BDL or UUO procedures determined by picrosirius red staining and immuno‐detection (Figure 7A‐D). Expression of α‐SMA using immunoblotting and mRNA expression of fibrogenesis markers as A*cta2*, Ccn2, *Col1a1,* and *Col3a1* displayed similar levels after BDL and UUO between HMGB1^Flox^ and HMGB1^ΔMac^ mice (Figure 7E‐H, Figure S2G‐H). Interestingly, most of the inflammatory markers *Emr1, Tnfα*, *Il‐1b,* and *Il‐6 *were decreased in HMGB1^ΔMac^ compared to HMGB1^Flox^ mice (Figure 7E‐H; Figure S2G,H) although no functional incidences have been emphasized. Finally, compared to sham‐operated mice, BDL induced a pronounced liver injury (Figure S6A,B) but there were no differences in serum ALT or AST levels between HMGB1^Flox^ and HMGB1^ΔMac^ mice. In parallel, BDL and UUO procedures provoked a marked increase of circulating levels of HMGB1 (Figure S6C), albeit not significant, compared to sham‐operated animals, and operated HMGB1^Flox^ and HMGB1^ΔMac^ mice displayed comparable amount of serum HMGB1 levels (Figure S6C). Altogether these results suggest that, despite a stronger level of fibrosis compared to room temperature conditions, thermoneutral housing did not reveal any role of macrophage‐specific HMGB1 in fibrosis progression. Therefore, conclusions drawn from room temperature experiments were fully confirmed under thermoneutral conditions.

## 4 DISCUSSION

In the expanding field of sterile inflammation biology, many open questions remain in regard to the effects of alarmin/DAMPs in acute inflammation, tissue regeneration, scarring, and fibrosis. Here, using conditional deletion of HMGB1 protein specifically in macrophages, we clearly demonstrate that macrophage‐derived HMGB1 protein does not play a key role in fibrogenesis in three different organs using a wide range of well‐established models in vivo. In contrast, a recent study by Ge et  al, using macrophage‐specific genetic ablation (LysM‐CRE) or neutralization strategies demonstrates that macrophage and hepatocyte‐derived HMGB1 rather participates in liver fibrosis.^41^ Employing cell‐specific knockout of HMGB1 in macrophages in vivo, our report questions the contribution of a prototypical DAMP or pro‐inflammatory cytokine HMGB1 in the pathogenesis of fibrosis. The opposite conclusions of both studies might be due to the use of different mice. While Ge et  al used a conditional deletion of HMGB1 exons 2‐3, we employed mice expressing floxed exons 2‐4. It is hard to fully explain this obvious discrepancy but the presence of the exon 4 in the study of Ge et  al may be a possible caveat as bioinformatics analysis tend to demonstrate the presence of an active open reading frame (ORF) even after CRE recombination, suggesting that exons 4 and 5 are still possibly translated and biologically active. These two different floxing strategies resulted in opposite results on HMGB1 and mitochondrial quality control and autophagy mechanism in vivo,^30^, ^42^ but similar outcomes in liver cancer progression^19^, ^43^ without really finding a rational molecular explanation.^44^, ^45^, ^46^ Further studies will be needed to decipher why both transgenic lines lead to similar conclusions in certain contexts and to opposite conclusions in specific settings.

The present work is clearly questioning the potential role of macrophage‐derived HMGB1 during fibrosis and its relevance in vivo. Numerous reports have been published in recent years, showing in vitro that innate immune cells might actively secrete HMGB1, after an inflammatory challenge such as LPS. In vivo, few reports suggesting that *macrophage‐derived* HMGB1 may be involved in acute inflammation diseases like in the colon or lungs.^47^, ^48^ But most of these in vivo studies used pharmacological agents such as ethyl‐pyruvate that has been identified as a potential blocker of the nuclear to cytoplasmic translocation^47^ typically observed in macrophages cell lines. In the meantime, other publications have demonstrated that epithelial cells might be capable of an active secretion of HMGB1 such as adipocytes, skin fibroblasts, or colon cancer cells^49^, ^50^, ^51^ implying that ethyl‐pyruvate may also prevent active secretion in these epithelial cells and would then not be specific of macrophages, undermining conclusions drawn from previous in vivo works using ethyl pyruvate. In this context, the in vivo role of macrophage‐derived HMGB1 is still elusive, and only cell‐specific deletion strategy might help to decipher precisely which processes are driven by macrophage‐derived HMGB1. To the best of our knowledge, only a few studies have been already published using a genetic model of macrophage‐specific invalidation of HMGB1. Among them, the work from Huebener et al demonstrated using two different CRE lines (MX1‐Cre and VAV1‐Cre) targeting bone marrow‐derived cells, that circulating levels of HMGB1 were severely blunted after an LPS challenge in vivo,^52^ meanwhile Yanai et  al, using LysM‐Cre deleter targeting more specifically macrophages, showed no changes in HMGB1 circulating levels after the same LPS stimulation.^53^ Taken together these results tend to suggest that macrophages might be marginally involved, as opposed to in vitro studies, in active secretion process of HMGB1 after an LPS challenge questioning the relevance of HMGB1 active secretion by macrophages in vivo. It is possible that LPS is substantially different from stimuli encountered by macrophages during fibrogenic challenges such as CCL_4_ injections. But while we noted that HMGB1 circulating and liver levels were increased during all fibrogenic models used in our study, we found no changes in HMGB1 levels in plasma (Figure S7A‐E), but a slight decreases in fibrotic livers (Figure S7F,G) harvested from HMGB1^ΔMac ^compared to HMGB1^Flox^ mice supporting one more time a marginal contribution of macrophage‐derived HMGB1 in the whole circulating pool but a rather more significant role at the tissue level in the liver after CCL_4 _injection or following BDL (Figure S7F,G).

Despite being the best possible approach in vivo, HMGB1 cell‐specific deletion may also have potential caveats that need to be considered. As now well established, HMGB1 is within the cell, an abundant nuclear factor conserved among all eukaryotic cells, which regulates chromatin conformation and gene regulation and once outside the cells, a danger signal promoting inflammatory reactions. By deleting HMGB1 in the macrophages, we de facto blunted both intra and extracellular pools of HMGB1. And in their report, Yanai et  al stated that HMGB1 macrophage‐specific deletion may have altered intracellular homeostasis and notably autophagy pathways,^53^ which would have serious consequences on macrophages physiology, polarizing them toward a pro‐inflammatory phenotype with notably a higher capacity to secrete IL1β and IL‐18 and promoting eventually macrophage cell death.^53^ In this context, we cannot rule out that deletion of *Hmgb1* gene in macrophages may have altered macrophages function in our settings. Thus, we could hypothesize that generating such confounding factors (modifying macrophage global function and survival) could prevent us to properly assess the sole HMGB1 extracellular function in vivo and possible impact on fibrogenesis. Only sophisticated genetic strategies could help to discriminate between the role in macrophages of HMGB1 as a nuclear factor in one hand and as a secreted factor in the other hand.

Despite unexpected results on macrophage‐derived HMGB1 and fibrogenesis, our study does not question the overall role of DAMPs in fibrogenesis. Several publications demonstrated using well‐characterized DAMPs receptors, belonging to the pattern recognition receptor (PRR) family, that DAMPs and sterile inflammation players are definitely driving tissue scarring and fibrogenesis. Reports addressing potential HMGB1 receptors involvement in fibrogenesis clearly demonstrate that PRRs such as toll‐like receptors (TLRs) or receptor for advanced glycation end products (RAGE) are causally connected to tissue scarring in the lungs, kidney, heart, or liver. TLR4 knockout mice are protected against fibrogenesis upon chemically or surgically induced fibrosis in the liver,^34^ heart,^54^ and lungs,^55^ TLR2 knockout mice are preserved toward cardiac fibrosis,^56^ and TLR9 has been involved in liver fibrosis.^57^, ^58^ And RAGE has also been connected to fibrogenesis in the lungs^59^, ^60^ and kidneys.^61^ Thus, there is a consistent body of literature demonstrating that PRRs such as TLRs or RAGE are involved in fibrogenesis, therefore suggesting that DAMPs or other types of ligands besides HMGB1, may exert a pro‐fibrotic activity.^62^ In this context, HMGB1 certainly acts as DAMP in certain conditions or as an alarmin in other conditions depending on the cell source. Considering the complexity of sterile inflammation response, with such a variety of ligands and receptors, one could hypothesize that initiation of an inflammatory or pro‐fibrotic response may depend on an unknown threshold of necrosis, type of tissue injury combined with micro‐environmental conditions—not yet identified—which could affect and influence the channeling toward no response, or inflammatory and/or fibrotic response. Further in vivo work is needed, using sophisticated genetic tools, to clearly assess how on a molecular level, how nuclear and secreted HMGB1 may drive regeneration, inflammation, or fibrosis and to identify which cell source is specifically involved in each biological pathway.

## CONFLICT OF INTEREST

The authors declare no conflicts of interest.

## AUTHOR CONTRIBUTIONS

J. Personnaz and E. Piccolo designed research, performed experiments, analyzed data, and wrote the paper; M. Branchereau, A. Filliol, R. Paccoud, E. Moreau, D. Calise, E. Riant and C. Heymes performed experiments and analyzed data, P. Gourdy generously provided LysM‐CRE mice; R.F. Schwabe kindly provided HMGB1‐floxed mice, designed experiments, and revised the manuscript; C. Dray and P. Valet provided fundings and revised the manuscript; J.‐P. Pradère conceived the original hypothesis, designed all experiments, performed experiments, analyzed data, wrote the manuscript, provided fundings, and supervised the project.

## References

[1] Mack M . Inflammation and fibrosis. Matrix Biol. 2018;68-69:106-121. 29196207 10.1016/j.matbio.2017.11.010

[2] Weiskirchen R , Weiskirchen S , Tacke F . Organ and tissue fibrosis: molecular signals, cellular mechanisms and translational implications. Mol Aspects Med. 2018. S0098-2997(18)30038-4. 10.1016/j.mam.2018.06.003 29958900

[3] Vannella KM , Wynn TA . Mechanisms of organ injury and repair by macrophages. Annu Rev Physiol. 2017;79:593‐617. 27959618 10.1146/annurev-physiol-022516-034356

[4] Smigiel KS , Parks WC . Macrophages, wound healing, and fibrosis: recent insights. Curr Rheumatol Rep. 2018;20:17. 29550962 10.1007/s11926-018-0725-5

[5] Thannickal VJ , Zhou Y , Gaggar A , Duncan SR . Fibrosis: ultimate and proximate causes. J Clin Invest. 2014;124:4673‐4677. 25365073 10.1172/JCI74368 PMC4347226

[6] Lee J‐M , Yoshida M , Kim M‐S , et al. Involvement of alveolar epithelial cell necroptosis in IPF pathogenesis. Am J Respir Cell Mol Biol. 2018;59:215-224. rcmb.2017‐0034OC. 29444413 10.1165/rcmb.2017-0034OC

[7] Zhang W , Lavine KJ , Epelman S , et al. Necrotic myocardial cells release damage‐associated molecular patterns that provoke fibroblast activation in vitro and trigger myocardial inflammation and fibrosis in vivo. J Am Heart Assoc. 2015;4:e001993‐e001993. 26037082 10.1161/JAHA.115.001993 PMC4599537

[8] Vénéreau E , Ceriotti C , Bianchi ME . DAMPs from cell death to new life. Front Immunol. 2015;6:422. 26347745 10.3389/fimmu.2015.00422 PMC4539554

[9] Andersson U , Yang H , Harris H . High‐mobility group box 1 protein (HMGB1) operates as an alarmin outside as well as inside cells. Semin Immunol. 2018;38:40‐48. 29530410 10.1016/j.smim.2018.02.011

[10] Kang R , Chen R , Zhang Q , et al. HMGB1 in health and disease. Mol Aspects Med. 2014;40:1‐116. 25010388 10.1016/j.mam.2014.05.001 PMC4254084

[11] Dear JW , Clarke JI , Francis B , et al. Risk stratification after paracetamol overdose using mechanistic biomarkers: results from two prospective cohort studies. Lancet Gastroenterol Hepatol. 2018;3:104‐113. 29146439 10.1016/S2468-1253(17)30266-2 PMC5777094

[12] Venereau E , De Leo F , Mezzapelle R , Careccia G , Musco G , Bianchi ME . HMGB1 as biomarker and drug target. Pharmacol Res. 2016;111:534‐544. 27378565 10.1016/j.phrs.2016.06.031

[13] Andersson U , Tracey KJ . HMGB1 is a therapeutic target for sterile inflammation and infection. Annu Rev Immunol. 2011;29:139‐162. 21219181 10.1146/annurev-immunol-030409-101323 PMC4536551

[14] Andersson U , Wang H , Palmblad K , et al. High mobility group 1 protein (HMG‐1) stimulates proinflammatory cytokine synthesis in human monocytes. J Exp Med, Rockefeller University Press, 2000;192:565‐570. 10952726 10.1084/jem.192.4.565 PMC2193240

[15] Gil M , Kim YK , Hong SB , Lee, KJ . Naringin decreases TNF‐α and HMGB1 release from LPS‐stimulated macrophages and improves survival in a CLP‐induced sepsis mice. Mukhopadhyay P, editor. PLoS ONE, Public Library of Science. 2016;11:e0164186. 27716835 10.1371/journal.pone.0164186 PMC5055320

[16] El Gazzar M . HMGB1 modulates inflammatory responses in LPS‐activated macrophages. Inflamm Res. 2007;56:162‐167. 17522814 10.1007/s00011-006-6112-0

[17] Gardella S , Andrei C , Ferrera D , et al. The nuclear protein HMGB1 is secreted by monocytes via a non‐classical, vesicle‐mediated secretory pathway. EMBO Rep. 2002;3:995‐1001. 12231511 10.1093/embo-reports/kvf198 PMC1307617

[18] Tirone M , Tran NL , Ceriotti C , et al. High mobility group box 1 orchestrates tissue regeneration via CXCR18. J Exp Med. 2018;215:303‐318. 29203538 10.1084/jem.20160217 PMC5748844

[19] Hernandez C , Huebener P , Pradere J‐P , Antoine DJ , Friedman RA , Schwabe RF . HMGB1 links chronic liver injury to progenitor responses and hepatocarcinogenesis. J Clin Invest. 2018;128:2436‐2451. 29558367 10.1172/JCI91786 PMC5983315

[20] Khambu B , Huda N , Chen X , et al. HMGB1 promotes ductular reaction and tumorigenesis in autophagy‐deficient livers. J Clin Invest. 2018;128:2419‐2435. 29558368 10.1172/JCI91814 PMC5983330

[21] Lee G , Espirito Santo AI , Zwingenberger S , et al. Fully reduced HMGB1 accelerates the regeneration of multiple tissues by transitioning stem cells to G_Alert_ . Proc Natl Acad Sci. 2018;115:E4463‐E4472. 29674451 10.1073/pnas.1802893115 PMC5949009

[22] Wu R‐N , Yu T‐Y , Zhou J‐C , et al. Targeting HMGB1 ameliorates cardiac fibrosis through restoring TLR2‐mediated autophagy suppression in myocardial fibroblasts. Int J Cardiol. 2018;267:156‐162. 29957254 10.1016/j.ijcard.2018.04.103

[23] Hamada N , Maeyama T , Kawaguchi T , et al. The role of high mobility group box1 in pulmonary fibrosis. Am J Respir Cell Mol Biol. 2008;39:440‐447. 18441281 10.1165/rcmb.2007-0330OC

[24] Tian S , Zhang L , Tang J , Guo X , Dong K , Chen S‐Y . HMGB1 exacerbates renal tubulointerstitial fibrosis through facilitating M1 macrophage phenotype at the early stage of obstructive injury. Am J Physiol Renal Physiol. 2015;308:F69‐75. 25377911 10.1152/ajprenal.00484.2014 PMC4281691

[25] Troeger JS , Mederacke I , Gwak G‐Y , et al. Deactivation of hepatic stellate cells during liver fibrosis resolution in mice. Gastroenterology. 2012;143:1073-1083.e22. 22750464 10.1053/j.gastro.2012.06.036 PMC3848328

[26] Pradere J‐P , Kluwe J , De Minicis S , et al. Hepatic macrophages but not dendritic cells contribute to liver fibrosis by promoting the survival of activated hepatic stellate cells in mice. Hepatology. 2013;58:1461-1473. 23553591 10.1002/hep.26429 PMC3848418

[27] Chevalier RL , Forbes MS , Thornhill BA . Ureteral obstruction as a model of renal interstitial fibrosis and obstructive nephropathy. Kidney Int. 2009;75:1145-1152. 19340094 10.1038/ki.2009.86

[28] Bisserier M , Berthouze‐Duquesnes M , Breckler M , et al. Carabin protects against cardiac hypertrophy by blocking calcineurin, Ras, and Ca^2+^/calmodulin‐dependent protein kinase II signaling. Circulation. 2015;131:390‐400. 25369805 10.1161/CIRCULATIONAHA.114.010686

[29] Clausen BE , Burkhardt C , Reith W , Renkawitz R , Förster I . Conditional gene targeting in macrophages and granulocytes using LysMcre mice. Transgenic Res. 1999;8:265–277. 10621974 10.1023/a:1008942828960

[30] Huebener P , Gwak G‐Y , Pradere J‐P , et al. High‐mobility group box 1 is dispensable for autophagy, mitochondrial quality control, and organ function in vivo. Cell Metab. 2014;19:539‐547. 24606906 10.1016/j.cmet.2014.01.014 PMC4099361

[31] Arriazu E , Ge X , Leung T‐M , et al. Signalling via the osteopontin and high mobility group box‐1 axis drives the fibrogenic response to liver injury. Gut. 2017;66:1123‐1137. 26818617 10.1136/gutjnl-2015-310752 PMC5532463

[32] He Q , Fu Y , Ding X , et al. High‐mobility group box 1 induces endoplasmic reticulum stress and activates hepatic stellate cells. Lab Investig. 2018;98:1200‐1210. 29959419 10.1038/s41374-018-0085-9

[33] Duffield JS , Forbes SJ , Constandinou CM , et al. Selective depletion of macrophages reveals distinct, opposing roles during liver injury and repair. J Clin Invest. 2005;115:56‐65. 15630444 10.1172/JCI22675 PMC539199

[34] Seki E , De Minicis S , Österreicher CH , et al. TLR4 enhances TGF‐β signaling and hepatic fibrosis. Nat Med. 2007;13:1324‐1332. 17952090 10.1038/nm1663

[35] Xia Y , Lee K , Li N , Corbett D , Mendoza L , Frangogiannis NG . Characterization of the inflammatory and fibrotic response in a mouse model of cardiac pressure overload. Histochem Cell Biol. 2009;131:471‐481. 19030868 10.1007/s00418-008-0541-5 PMC2782393

[36] Giles DA , Moreno‐Fernandez ME , Stankiewicz TE , et al. Thermoneutral housing exacerbates nonalcoholic fatty liver disease in mice and allows for sex‐independent disease modeling. Nat Med, Nature Publishing Group. 2017;23:829‐838. 28604704 10.1038/nm.4346 PMC5596511

[37] Ganeshan K , Chawla A . Warming the mouse to model human diseases. Nat Rev Endocrinol. 2017;13:458‐465. 28497813 10.1038/nrendo.2017.48 PMC5777302

[38] Tian XY , Ganeshan K , Hong C , et al. Thermoneutral housing accelerates metabolic inflammation to potentiate atherosclerosis but not insulin resistance. Cell Metab, Elsevier. 2016;23:165‐178. 26549485 10.1016/j.cmet.2015.10.003 PMC4715491

[39] Wick G , Backovic A , Rabensteiner E , Plank N , Schwentner C , Sgonc R . The immunology of fibrosis: innate and adaptive responses. Trends Immunol, Europe PMC Funders. 2010;31:110‐119. 20106721 10.1016/j.it.2009.12.001 PMC3292796

[40] Koyama Y , Brenner DA . Liver inflammation and fibrosis. J Clin Invest, American Society for Clinical Investigation. 2017;127:55‐64. 28045404 10.1172/JCI88881 PMC5199698

[41] Ge X , Arriazu E , Magdaleno F , et al. High mobility group box‐1 drives fibrosis progression signaling via the receptor for advanced glycation end‐products in mice. Hepatology. 2018;68:2380‐2404. 29774570 10.1002/hep.30093 PMC6240507

[42] Tang D , Kang R , Livesey KM , et al. High‐mobility group box 1 is essential for mitochondrial quality control. Cell Metab. 2011;13:701‐711. 21641551 10.1016/j.cmet.2011.04.008 PMC3293110

[43] Chen R , Zhu S , Fan X‐G , et al. High mobility group protein B1 controls liver cancer initiation through yes‐associated protein ‐dependent aerobic glycolysis. Hepatology. 2018;67:1823‐1841. 29149457 10.1002/hep.29663 PMC5906197

[44] Sun X , Tang D . Hepatocyte‐specific *Hmgb1* deletion. Autophagy. 2015;11:1189‐1191. 26043873 10.1080/15548627.2015.1054595 PMC4590590

[45] Huebener P , Gwak GY , Schwabe RF . Comment on: HMGB1‐dependent and ‐independent autophagy. Autophagy. 2015;11:1187‐1188. 26121576 10.1080/15548627.2015.1054593 PMC4590679

[46] Sun X , Tang D . HMGB1‐dependent and ‐independent autophagy. Autophagy. 2014;10:1873‐1876. 25126737 10.4161/auto.32184 PMC4198373

[47] Davé SH , Tilstra JS , Matsuoka K , et al. Ethyl pyruvate decreases HMGB1 release and ameliorates murine colitis. J Leukoc Biol. 2009;86:633‐643. 19454652 10.1189/jlb.1008662 PMC2735284

[48] Entezari M , Javdan M , Antoine DJ , et al. Inhibition of extracellular HMGB1 attenuates hyperoxia‐induced inflammatory acute lung injury. Redox Biol. 2014;2:314‐322. 24563849 10.1016/j.redox.2014.01.013 PMC3926109

[49] Shimizu T , Yamakuchi M , Biswas KK , et al. HMGB1 is secreted by 3T3‐L1 adipocytes through JNK signaling and the secretion is partially inhibited by adiponectin. Obesity (Silver Spring). 2016;24:1913‐1921. 27430164 10.1002/oby.21549

[50] Lee H , Park M , Shin N , et al. High mobility group box‐1 is phosphorylated by protein kinase C zeta and secreted in colon cancer cells. Biochem Biophys Res Commun. 2012;424:321‐326. 22750245 10.1016/j.bbrc.2012.06.116

[51] Wang L , He L , Bao G , He X , Fan S , Wang, H . Ionizing radiation induces HMGB1 cytoplasmic translocation and extracellular release. Guo Ji Fang She Yi Xue He Yi Xue Za Zhi. 2016;40:91‐99. 27331198 PMC4911189

[52] Huebener P , Pradere J‐P , Hernandez C , et al. The HMGB1/RAGE axis triggers neutrophil‐mediated injury amplification following necrosis. J Clin Invest. 2015;125:539‐550. 25562324 10.1172/JCI76887 PMC4319429

[53] Yanai H , Matsuda A , An J , et al. Conditional ablation of HMGB1 in mice reveals its protective function against endotoxemia and bacterial infection. Proc Natl Acad Sci. 2013;110:20699‐20704. 24302768 10.1073/pnas.1320808110 PMC3870753

[54] Li XX , Jiang DY , Huang XX , Guo SL , Yuan W , Dai HP . Toll‐like receptor 4 promotes fibrosis in bleomycin‐induced lung injury in mice. Genet Mol Res. 2015;14:17391‐17398. 26782380 10.4238/2015.December.21.8

[55] Dong R , Wang Z , Zhao C , et al. Toll‐like receptor 4 knockout protects against isoproterenol‐induced cardiac fibrosis: the role of autophagy. J Cardiovasc Pharmacol Ther. 2015;20:84‐92. 24950765 10.1177/1074248414539564

[56] Wang L , Li Y‐L , Zhang C‐C , et al. Inhibition of Toll‐like receptor 2 reduces cardiac fibrosis by attenuating macrophage‐mediated inflammation. Cardiovasc Res. 2014;101:383‐392. 24259498 10.1093/cvr/cvt258

[57] Watanabe A , Hashmi A , Gomes DA , et al. Apoptotic hepatocyte DNA inhibits hepatic stellate cell chemotaxis via toll‐like receptor 9. Hepatology. 2007;46:1509‐1518. 17705260 10.1002/hep.21867

[58] Gäbele E , Mühlbauer M , Dorn C , et al. Role of TLR9 in hepatic stellate cells and experimental liver fibrosis. Biochem Biophys Res Commun. 2008;376:271‐276. 18760996 10.1016/j.bbrc.2008.08.096

[59] Englert JM , Hanford LE , Kaminski N , et al. A role for the receptor for advanced glycation end products in idiopathic pulmonary fibrosis. Am J Pathol. 2008;172:583‐591. 18245812 10.2353/ajpath.2008.070569 PMC2258251

[60] Ramsgaard L , Englert JM , Tobolewski J , et al. The role of the receptor for advanced glycation end‐products in a murine model of silicosis. Morty RE, editor. PLoS ONE. 2010;5:e9604. 20333255 10.1371/journal.pone.0009604 PMC2841632

[61] Gasparitsch M , Arndt A‐K , Pawlitschek F , et al. RAGE‐mediated interstitial fibrosis in neonatal obstructive nephropathy is independent of NF‐κB activation. Kidney Int. 2013;84:911‐919. 23677242 10.1038/ki.2013.171

[62] Chen L , Li J , Zhang J , et al. S100A4 promotes liver fibrosis via activation of hepatic stellate cells. J Hepatol. 2015;62:156‐164. 25111176 10.1016/j.jhep.2014.07.035

