# Supplementary figures and images for: Macrophage‐derived HMGB1 is dispensable for tissue fibrogenesis
Source: FASEB Bioadv. 2019 Feb 12;1(4):227–45. doi: 10.1096/fba.2018-00035 (PMC6996376; doi:10.1096/fba.2018-00035)

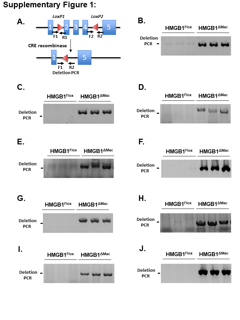

Supplement: Supplementary file 1 [file FBA2-1-227-s001.tif]
