# Supplementary material for: Macrophage‐derived HMGB1 is dispensable for tissue fibrogenesis
Source: FASEB Bioadv. 2019 Feb 12;1(4):227–45. doi: 10.1096/fba.2018-00035 (PMC6996376; doi:10.1096/fba.2018-00035)

Supplementary Figure 1:

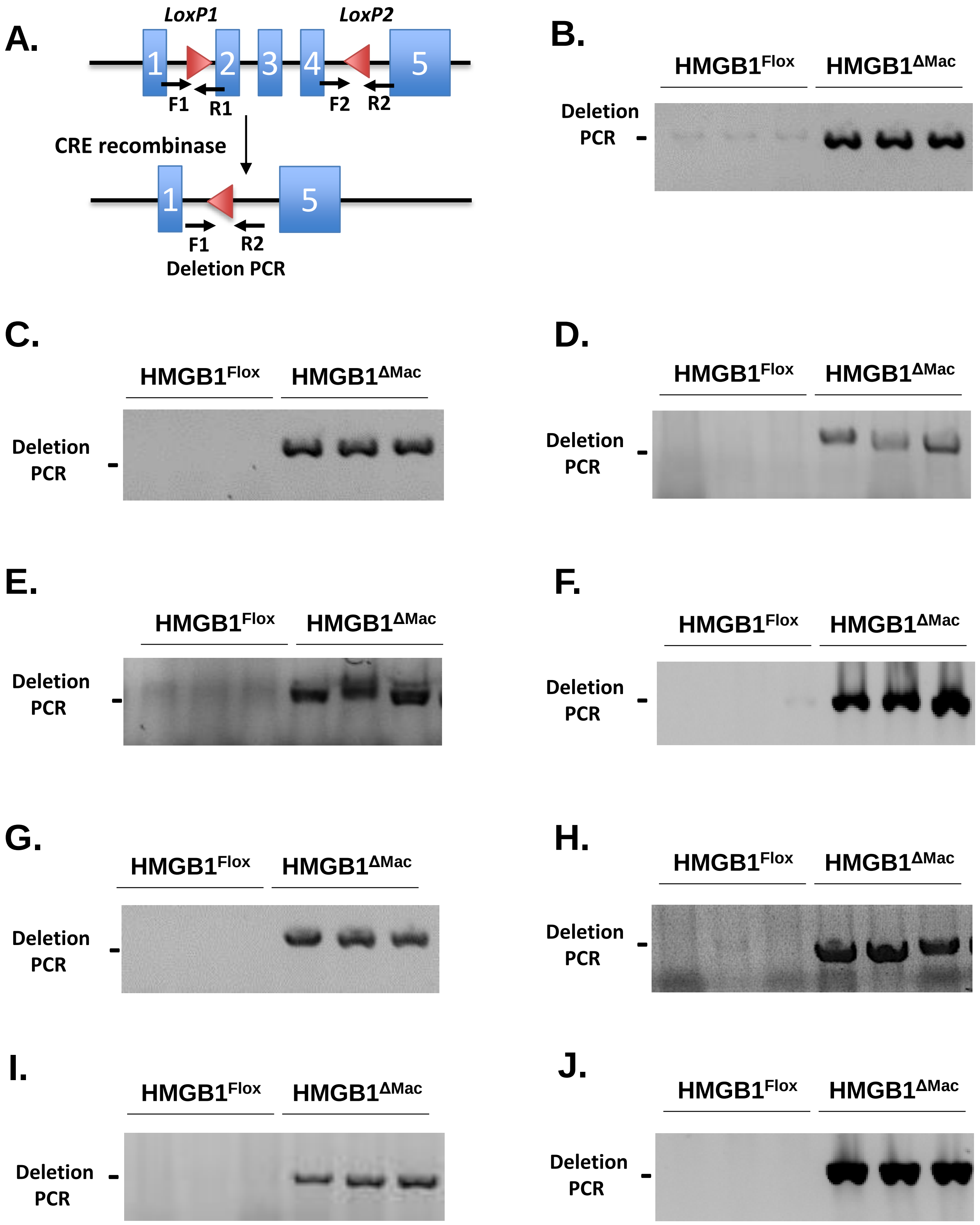

Supplementary Figure 2:

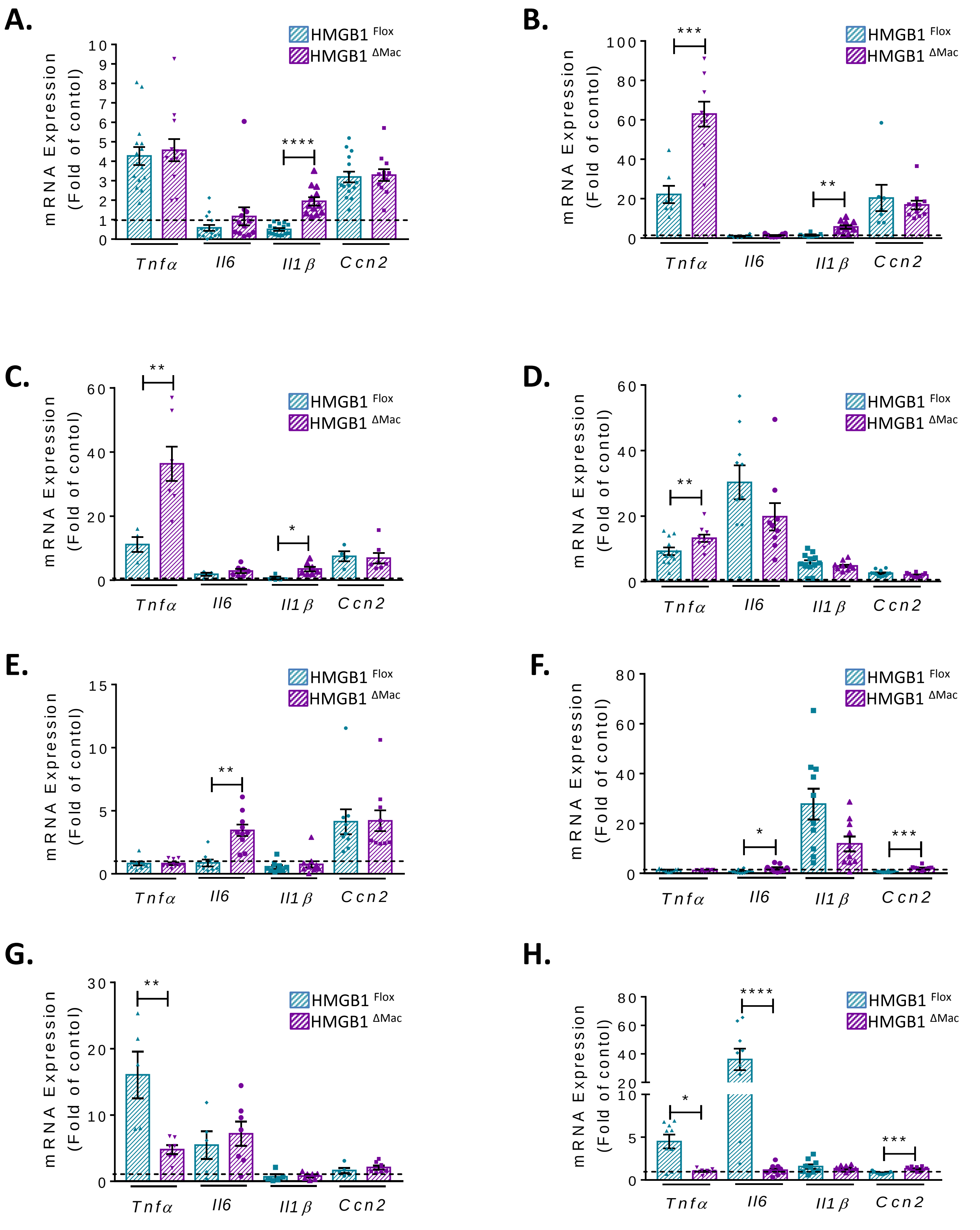

Supplementary Figure 3:

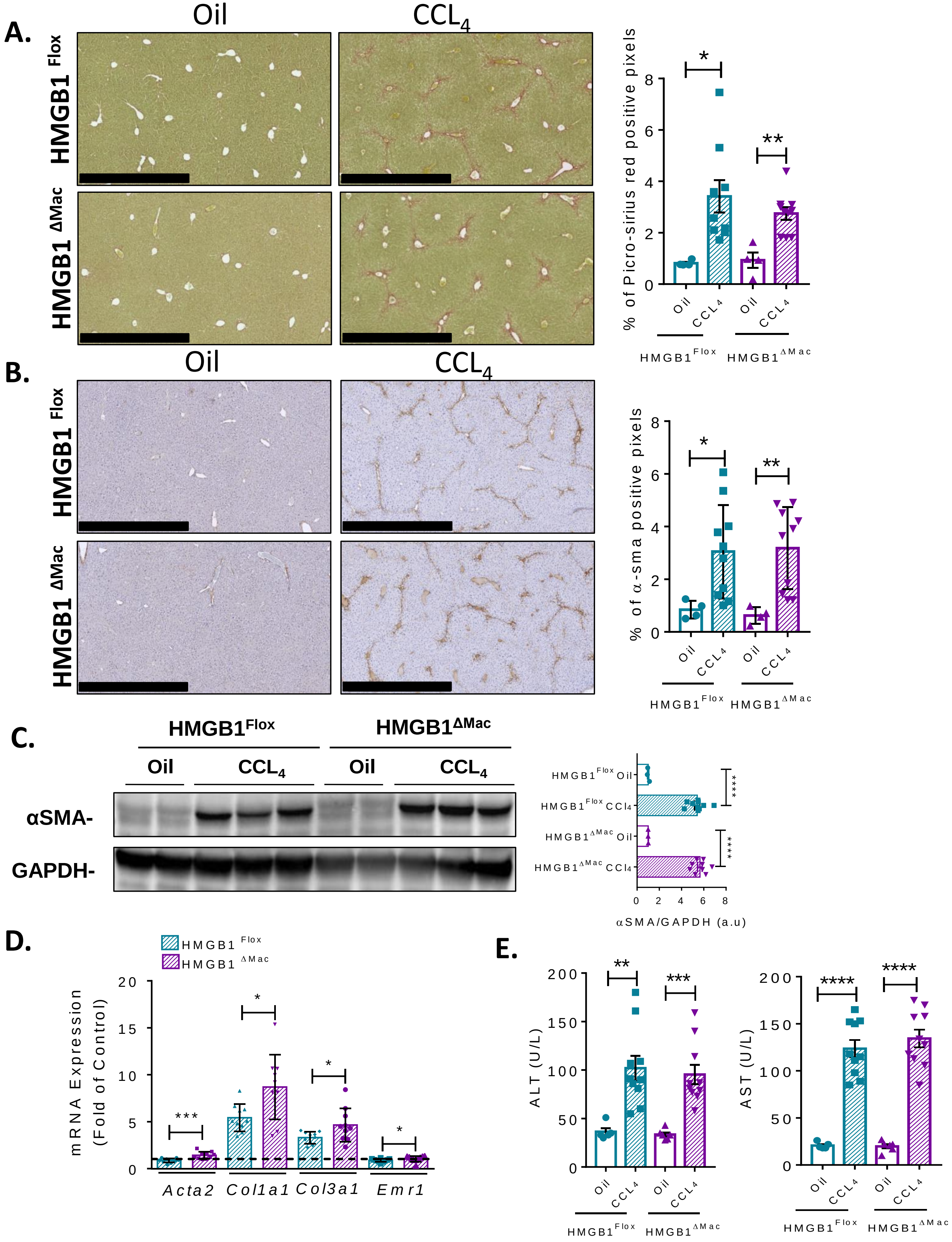

Supplementary Figure 4:

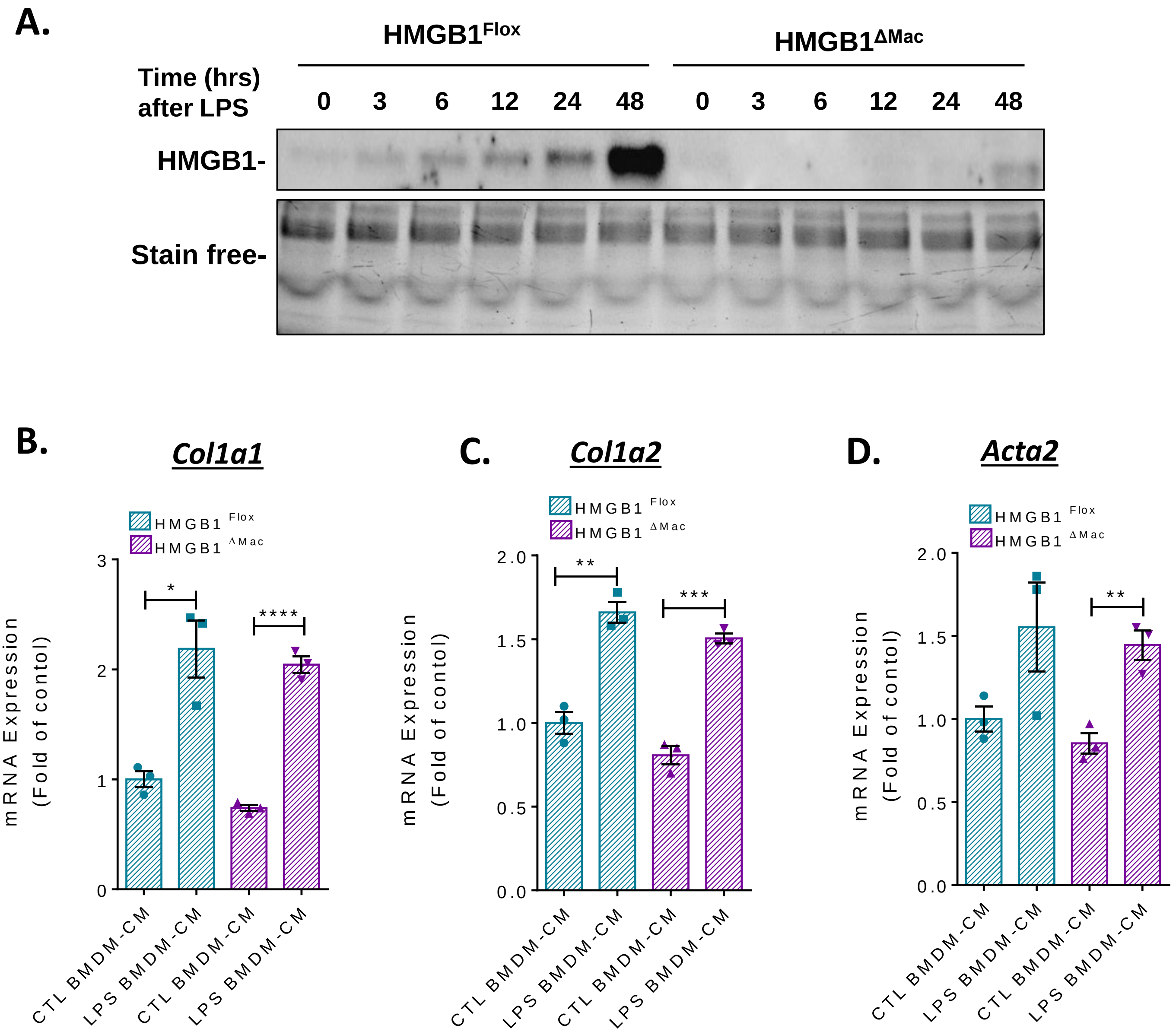

Supplementary Figure 5:

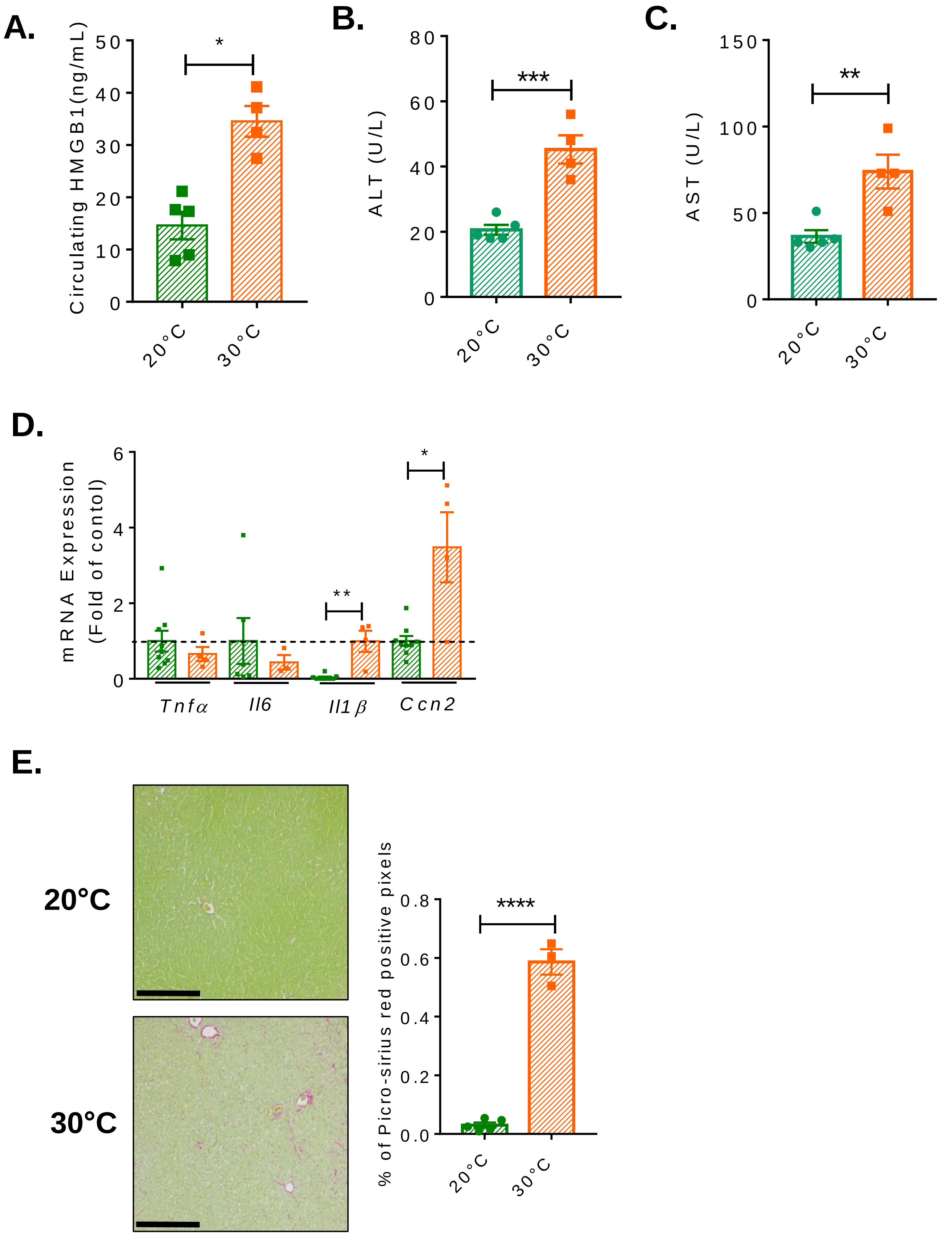

Supplementary Figure 6:

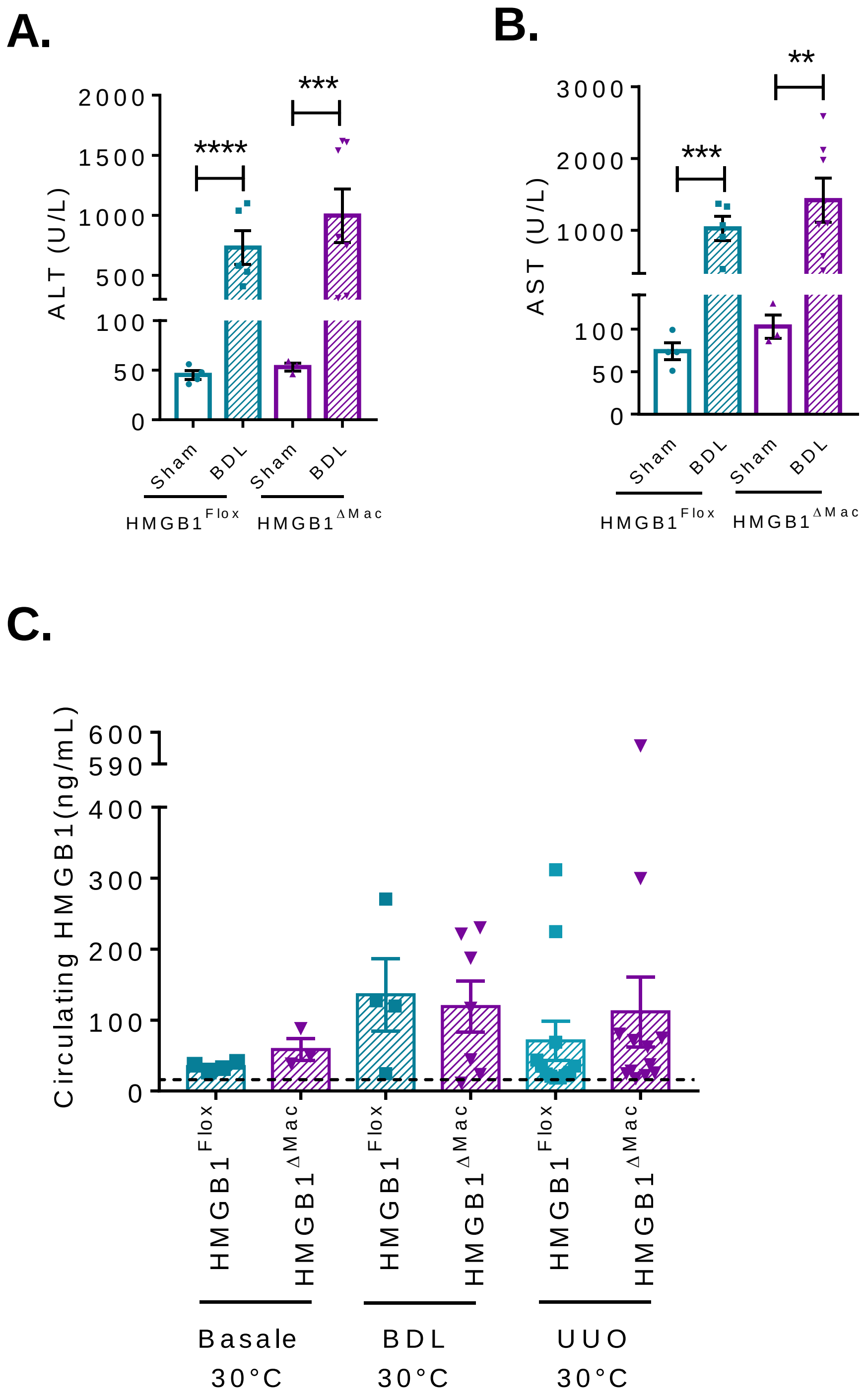

Supplementary Figure 7:

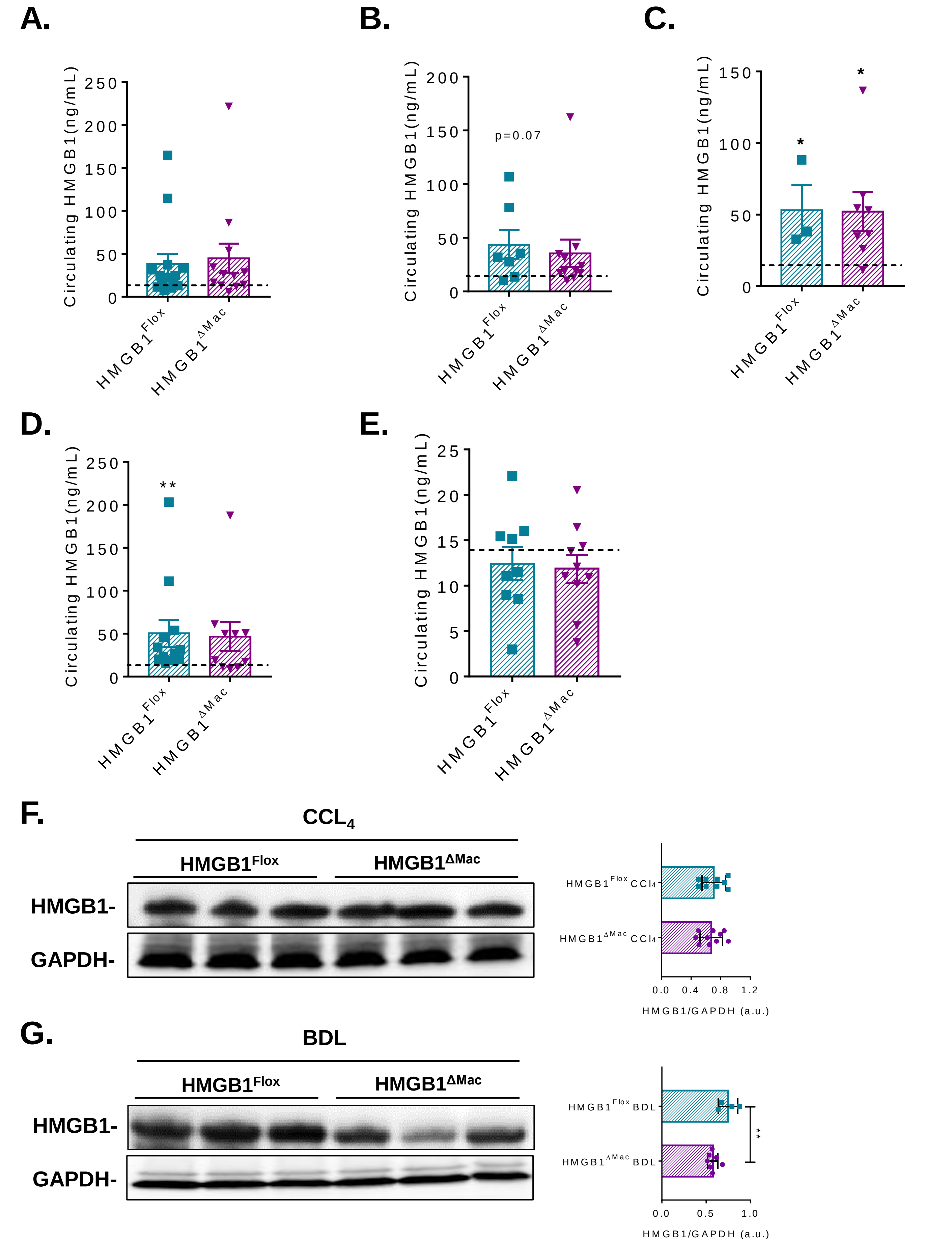

Supplement: Supplementary file 2 [file FBA2-1-227-s002.pdf]
