# Supplementary material for: Macrophage‐derived HMGB1 is dispensable for tissue fibrogenesis
Source: FASEB Bioadv. 2019 Feb 12;1(4):227–45. doi: 10.1096/fba.2018-00035 (PMC6996376; doi:10.1096/fba.2018-00035)
Supplement: Supplementary file 3 [file FBA2-1-227-s003.pdf]

Supplementary Figure 1: Deletion PCR in all mice cohorts of our study. PCR strategy used to determine the gene deletion after recombination generated by LysM-Cre recombinase using Forward primer F1 and Reverse R2 flanking remnant loxP site inserted between exon 1 and 5 (A). Representative pictures of deletion PCR from DNA extracted on whole liver on $HMGB1^{Flox}$ and $HMGB1^{\Delta Mac}$ under control conditions (B). Representative pictures of deletion PCR from DNA extracted on whole liver on $HMGB1^{Flox}$ and $HMGB1^{\Delta Mac}$ subjected to 8 injections of CCL_4 (C). Representative pictures of deletion PCR from DNA extracted on whole liver on $HMGB1^{Flox}$ and $HMGB1^{\Delta Mac}$ subjected to 18 injections of TAA (D). Representative pictures of deletion PCR from DNA extracted on whole liver on $HMGB1^{Flox}$ and $HMGB1^{\Delta Mac}$ subjected to 21 days of BDL (E). Representative pictures of deletion PCR from DNA extracted on whole kidney on $HMGB1^{Flox}$ and $HMGB1^{\Delta Mac}$ subjected to 7 days of UUO (F). Representative pictures of deletion PCR from DNA extracted on whole heart on $HMGB1^{Flox}$ and $HMGB1^{\Delta Mac}$ subjected to 28 days of TAC (G). Representative pictures of deletion PCR from DNA extracted on whole liver on $HMGB1^{Flox}$ and $HMGB1^{\Delta Mac}$ under control conditions and housed at 30°C (H). Representative pictures of deletion PCR from DNA extracted on whole liver on $HMGB1^{Flox}$ and $HMGB1^{\Delta Mac}$ housed at 30°C and subjected to 21 days of BDL (I). Representative pictures of deletion PCR from DNA extracted on whole kidney on $HMGB1^{Flox}$ and $HMGB1^{\Delta Mac}$ housed at 30°C and subjected to 7 days of UUO (J).

Supplementary Figure 2: qPCR analysis of inflammatory markers *Tnfα, Il-6* and *Il-1β* and fibrosis markers *Ccn2* in livers from $HMGB1^{Flox}$ and $HMGB1^{\Delta Mac}$ mice subjected to 8 injections of CCL_4 (A), 18 injections of TAA (B), BDL (C), UUO (D), TAC (E) and 4 injections of CCL_4 (F) or BDL (G) and UUO (H) at thermoneutral housing (dotted line indicates basal level). Statistical analysis was performed with Mann and Whitney test. Data are expressed as means ± SEM. n=5 in $HMGB1^{Flox}$/Sham group; n=5 in $HMGB1^{\Delta Mac}$/Sham group; n=12 in $HMGB1^{Flox}$/8xCCL_4 group; n=11 in $HMGB1^{\Delta Mac}$/8xCCL_4 group; n=6 in $HMGB1^{Flox}$/TAA group; n=11 in $HMGB1^{\Delta Mac}$/TAA group; n=3 in $HMGB1^{Flox}$/BDL group; n=7 in $HMGB1^{\Delta Mac}$/BDL group; n=12 in $HMGB1^{Flox}$/UUO group; n=10 in $HMGB1^{\Delta Mac}$/UUO group; n=9 in $HMGB1^{Flox}$/TAC group; n=10 in $HMGB1^{\Delta Mac}$/TAC group; n=4 in $HMGB1^{Flox}$/Sham group; n=4 in $HMGB1^{\Delta Mac}$/Sham group; n=10 in $HMGB1^{Flox}$/4xCCL_4 group; n=10 in $HMGB1^{\Delta Mac}$/4xCCL_4 group; n=4 in $HMGB1^{Flox}$/basal 30°C group; n=3 $HMGB1^{Mac}$/basal 30°C group. n=5 in $HMGB1^{Flox}$/BDL 30°C group; n=7 $HMGB1^{Mac}$/BDL 30°C group; n=10 in $HMGB1^{Flox}$/UUO 30°C group; n=10 $HMGB1^{Mac}$/UUO30°C group. *p<0,05, **p<0,01, ***p<0,001, ****p<0,001.

Supplementary Figure 3: Macrophage-specific deletion of HMGB1 does not modify liver fibrogenesis after 4xCCL_4 treatment. Representative pictures of picrosirius red staining of $HMGB1^{Flox}$ mice and $HMGB1^{\Delta Mac}$ of liver section and quantification of positive pixels per liver section. Scale bar: 500 µm (A). Representative pictures of immunohistochemical staining with an antibody against α-SMA and quantification of positive pixels per liver section. Scale bar: 500 µm (B). Liver extracts from $HMGB1^{Flox}$ mice and $HMGB1^{\Delta Mac}$ were analyzed by western blotting directed against α–SMA (C). Liver mRNA expression levels of classical fibrosis markers were detected using quantitative RT-qPCR, the dotted line indicates the baseline (D). Hepatic injury in $HMGB1^{Flox}$ mice and $HMGB1^{\Delta Mac}$ was determined by ALT (left panel) and AST (right panel) levels (E). Statistical analysis was performed using Mann and Whitney test. Data are expressed as means ± SEM. n=4 in $HMGB1^{Flox}$/oil group; n=4 in $HMGB1^{\Delta Mac}$/oil group; n=10 in $HMGB1^{Flox}$/4xCCL_4 group; n=10 in $HMGB1^{\Delta Mac}$/4xCCL_4 group. *p<0,05, **p<0,01, ***p<0,001, **** p<0,0001 versus oil/4xCCL_4.

Supplementary Figure 4: Macrophages contribute to hepatic stellate cells (HSC) activation but in an HMGB1-independent manner. Bone marrow-derived macrophages (BMDM) from $HMGB1^{Flox}$ mice and $HMGB1^{\Delta Mac}$ mice were treated with LPS (10ng/mL) for 90 minutes and HMGB1 levels in conditioned media (CM) were measured using western blot at the indicated time points. Loading control is displayed using Stain Free staining (A). Starved HSCs were cultured in the presence of CM from naïve or LPS-stimulated BMDM isolated from $HMGB1^{Flox}$ mice and $HMGB1^{\Delta Mac}$ mice, for 24 hours. mRNA expression of HSC activation markers such as *Col1a1*, *Col1a2* or *Acta2* was determined by RT-qPCR (B-D). Statistical analysis was performed using t-test. Data are expressed as means of triplicate of cultured HSC ± SEM. n=3 $HMGB1^{Flox}$/CTL; n=3 $HMGB1^{Flox}$/LPS, n=3 $HMGB1^{Mac}$/CTL, n=3 $HMGB1^{Mac}$/LPS representative of two independent experiments. *p<0,05, **p<0,01, ***p<0,001, **** p<0,0001 versus CTL vs LPS.

Supplementary Figure 5: Remarkable changes induced by thermoneutral housing. **A:** Comparison of HMGB1 circulating levels in wild-type mice housed at 20°C and 30°C determined by ELISA (A). Circulating ALT (B) and AST (C) levels, mRNA expression of inflammation and fibrosis markers such as *Tnfα*, *Il-6*, *Il-1β* and *Ccn2* using RT-qPCR analysis (D) in wild-type mice housed at 20°C and 30°C. Representative pictures of picrosirius red

staining of liver from wild-type mice housed at 20°C and 30°C (E). Scale bar 250um. Statistical analysis was performed with Mann and Whitney test. Data are expressed as means ± SEM. n=5 in 20°C group; n=4 in 30°C group. *p<0,05, **p<0,01, ***p<0,001, ****p<0,001 versus 20°C vs 30°C.

Supplementary Figure 6: Thermoneutral housing effects during BDL and UUO. Circulating ALT (A) and AST (B) levels in $HMGB1^{Flox}$ and $HMGB1^{\Delta Mac}$ mice subjected to BDL (A-B). HMGB1 circulating levels in $HMGB1^{Flox}$ and $HMGB1^{\Delta Mac}$ mice subjected to BDL and UUO, a dotted line indicates the basal level of HMGB1 circulating levels (C). Statistical analysis was performed with Mann and Whitney analysis. Data are expressed as means ± SEM. n=4 in $HMGB1^{Flox}$/basal group; n=3 $HMGB1^{Mac}$/basal group. n=5 in $HMGB1^{Flox}$/BDL group; n=7 $HMGB1^{Mac}$/BDL group; n=10 in $HMGB1^{Flox}$/UUO group; n=10 $HMGB1^{Mac}$/UUO group. *p<0,05, **p<0,01, ***p<0,001 versus 20°C vs 30°C.

Supplementary Figure 7: Circulating levels of HMGB1 during fibrogenesis. Serum levels of HMGB1 determined by ELISA assay, in $HMGB1^{Flox}$ and $HMGB1^{\Delta Mac}$ mice subjected to CCL4 treatment, a dotted line indicates the basal level (A). Serum levels determined by ELISA assay of HMGB1 in $HMGB1^{Flox}$ and $HMGB1^{\Delta Mac}$ mice subjected to TAA treatment, a dotted line indicates basal level (B). Serum levels of HMGB1 determined by ELISA assay, in $HMGB1^{Flox}$ and $HMGB1^{\Delta Mac}$ mice subjected to BDL, a dotted line indicates basal level (C). Serum levels of HMGB1 determined by ELISA assay, in $HMGB1^{Flox}$ and $HMGB1^{\Delta Mac}$ mice subjected to UUO, a dotted line indicates basal level (D). Serum levels of HMGB1 determined by ELISA assay, in $HMGB1^{Flox}$ and $HMGB1^{\Delta Mac}$ mice subjected to TAC, a dotted line indicates basal level (E). HMGB1 levels were analyzed using immunoblotting after CCL_4 injections (F) or following BDL (G) in liver lysates from $HMGB1^{Flox}$ and $HMGB1^{\Delta Mac}$ mice. Statistical analysis was performed with Mann and Whitney test. Data are expressed as means ± SEM. n=5 in $HMGB1^{Flox}$/Sham group; n=5 in $HMGB1^{\Delta Mac}$/Sham group; n=12 in $HMGB1^{Flox}$/CCL_4 group; n=11 in $HMGB1^{\Delta Mac}$/CCL_4 group; n=6 in $HMGB1^{Flox}$/TAA group; n=11 in $HMGB1^{\Delta Mac}$/TAA group; n=3 in $HMGB1^{Flox}$/BDL group; n=7 in $HMGB1^{\Delta Mac}$/BDL group; n=12 in $HMGB1^{Flox}$/UUO group; n=10 in $HMGB1^{\Delta Mac}$/UUO group; n=9 in $HMGB1^{Flox}$/TAC group; n=10 in $HMGB1^{\Delta Mac}$/TAC group. *p<0,05, **p<0,01, ***p<0,001 versus toxin/surgery vs vehicle/sham.
